# Particle atomic layer deposition

**DOI:** 10.1007/s11051-018-4442-9

**Published:** 2019-01-04

**Authors:** Alan W. Weimer

**Affiliations:** Chemical and Biological Engineering, University of Colorado, Boulder, CO 80309-0596 USA

**Keywords:** Atomic layer deposition, Particle ALD, Nanoparticle, Nanolayers, Coating

## Abstract

The functionalization of fine primary particles by atomic layer deposition (particle ALD) provides for nearly perfect nanothick films to be deposited conformally on both external and internal particle surfaces, including nanoparticle surfaces. Film thickness is easily controlled from several angstroms to nanometers by the number of self-limiting surface reactions that are carried out sequentially. Films can be continuous or semi-continuous. This review starts with a short early history of particle ALD. The discussion includes agitated reactor processing, both atomic and molecular layer deposition (MLD), coating of both inorganic and polymer particles, nanoparticles, and nanotubes. A number of applications are presented, and a path forward, including likely near-term commercial products, is given.

## Introduction

**Fig. 1:**
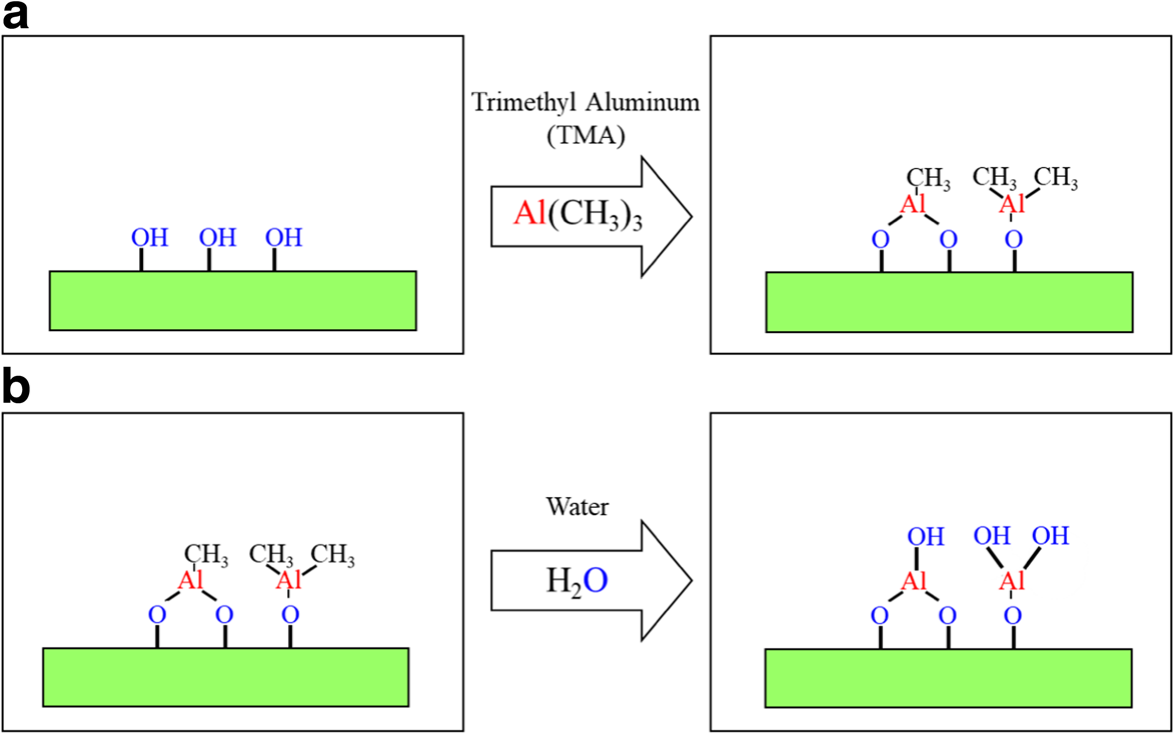
Binary reaction sequence for alumina ALD. Binary rxn: 2Al(CH_3_)_3_ + 3 H_2_O → Al_2_O_3_ + 6 CH_4_. A reaction: 2AlOH* + 2Al(CH_3_)_3_ → 2[Al-O-Al(CH_3_)_2_]* + 2CH_4_. B reaction: 2[Al-O-Al(CH_3_)_2_]* + 3H_2_O → Al_2_O_3_ + 2AlOH* + 4CH_4_. *Surface species

**Fig. 2:**
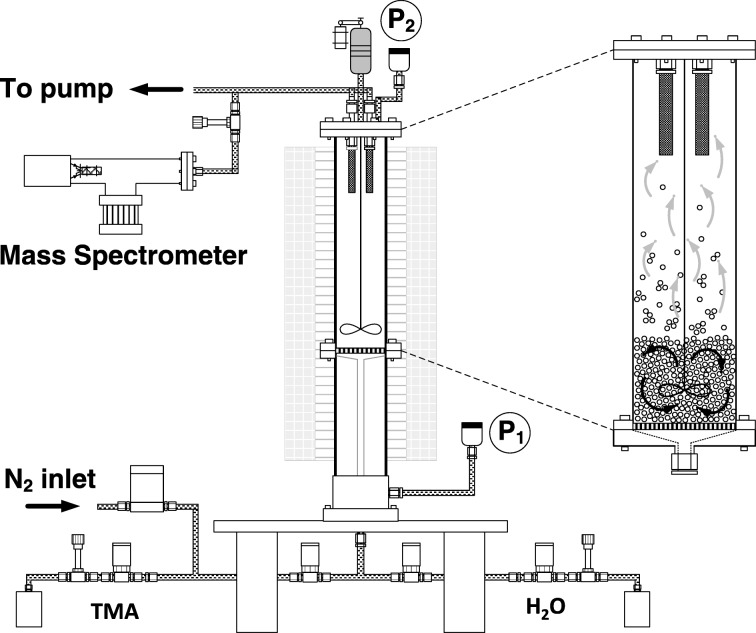
Fluidized bed particle ALD process (King et al. 2007)

**Fig. 3:**
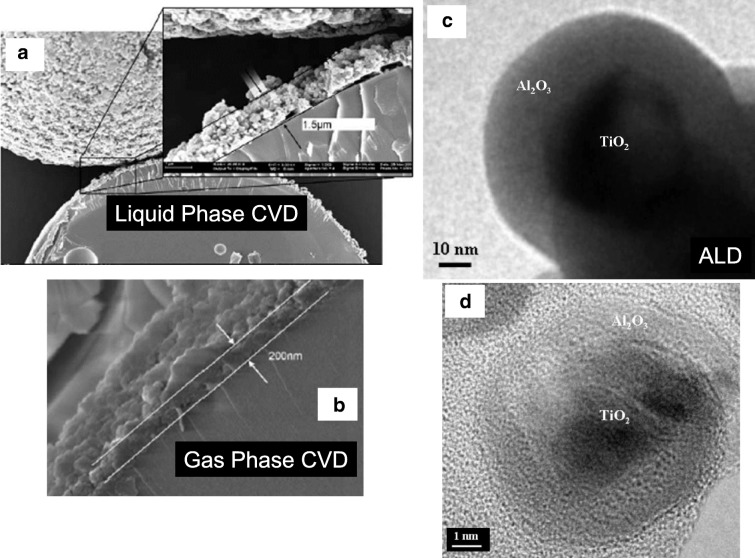
Comparison of CVD and ALD particle coatings: (**a**) liquid-phase aluminum CVD on 64 μm glass spheres (Czok and Werther, 2006); (**b**) gas-phase aluminum CVD on 64 μm glass spheres (Czok and Werther, 2006); (**c**) alumina ALD film on 40 nm TiO_2_ particle substrate (Hakim et al. 2007b); (**d**) alumina ALD film on 15 nm TiO_2_ particle substrate (Hakim et al. 2007b)

The conformal nanocoating of ultrafine dry primary particles, including nanoparticles with pin-hole-free films, is controlled to within several angstroms thick using atomic layer deposition (ALD), i.e., particle ALD. Atomic layer control is provided by self-limiting sequential surface reactions (separate saturating gas–solid reactions) in order to prevent non-desired gas-phase reactions between precursors (George 2010). Repeated sequential dosing of chemical precursors (Fig. 1) to coat particles is done using agitated particle reactors, such as fluidized beds (King et al. 2007) (Fig. 2). The thickness of the films is controlled by the number of sequential cycles that are repeated. Without sequential ALD dosing, gas-phase reactions occur. Nanoparticles that are generated in the gas phase (i.e., via chemical vapor deposition (CVD)) are then scavenged on the surface of the substrate particles forming porous films (Powell et al. 1997) (Fig. 3).

## General chemistry

Generically, a CVD reaction can be divided into successive surface reactions that occur solely on a particle surface, reacting surface functional groups, to define ALD. For example, to deposit aluminum oxide (Al_2_O_3_), the binary CVD reaction between trimethylaluminum (Al(CH_3_)_3_) (TMA) and water vapor (H_2_O) generates methane (CH_4_) as a byproduct according to Reaction (1).

1 $$ \mathrm{Binary}\ \mathrm{CVD}\ \mathrm{Reaction}:2\mathrm{Al}{\left({\mathrm{CH}}_3\right)}_3+3\ {\mathrm{H}}_2\mathrm{O}\to {\mathrm{Al}}_2{\mathrm{O}}_3+6{\mathrm{CH}}_4 $$

For ALD, the binary CVD Reaction (1) can be split into two successive surface reactions, shown in the subsequent texts. The asterisk (*) denotes a surface reaction, and so, if TMA and H_2_O are not present simultaneously (as is done in CVD), the reaction occurs entirely at the surface and no gas-phase reaction-producing nanoparticles occur. The sequential surface reactions A (2) and B (3) are then repeated (cycled) in order to grow an ultrathin and conformal film (Ott et al. 1997a, b) (Fig. 3c, d):

2 $$ \mathrm{A}\;\mathrm{reaction}:{\mathrm{AlOH}}^{\ast }+\mathrm{Al}{\left({\mathrm{CH}}_3\right)}_3\to \mathrm{A}\mathrm{l}-\mathrm{O}-\mathrm{Al}{{\left({\mathrm{CH}}_3\right)}_2}^{\ast }+{\mathrm{CH}}_4 $$

3 $$ \mathrm{B}\ \mathrm{reaction}:\mathrm{Al}-\mathrm{O}-\mathrm{Al}{{\left({\mathrm{CH}}_3\right)}_2}^{\ast }+1.5{\mathrm{H}}_2\mathrm{O}\to {\mathrm{AlO}}_{1.5}-{\mathrm{AlO}\mathrm{H}}^{\ast }+2{\mathrm{CH}}_4 $$

Deposition is controlled at the atomic level by self-limiting surface reactions (George et al. 1996). The process is independent of line of sight. Hence, uniform and conformal deposition will occur on high-aspect ratio porous structures or on particles in particle beds because the surface chemistry is self-passivating (Ott et al. 1997a, b). Precursors do not self-react; they only react with the functionalized surface produced by the reaction with the complementary precursor. Once the reaction is completed at one surface site, the reactants will continue to travel down the high-aspect ratio pore or convoluted path in the particle bed and reach the unreacted surface sites. Consequently, the deposition produced by each surface reaction only proceeds until no further active sites are accessible to the precursor on the substrate surface, making the deposition self-limiting. The thickness of the film is only dependent on the number of times the surface reactions are cycled, i.e., AB cycles. A review of ALD chemistry is given by George et al. (2000) and George (2010).

## Early history of particle ALD

ALD was pioneered in Finland in the early 1970s (Suntola and Jorma 1977). Particle ALD was pioneered in the late 1990s at the University of Colorado (George et al. 2003, 2004, 2005), and it is characterized by the ability to coat primary particles (not agglomerating them in the process), including nanoparticles with conformal films. The initial objectives for particle ALD were to deposit pinhole-free films as thin as possible in order to provide an environmental barrier coating (EBC) or to functionalize the particle surface for a specific application. A major effort was identifying a minimum film thickness for a barrier or to achieve a particular effect.

**Fig. 4:**
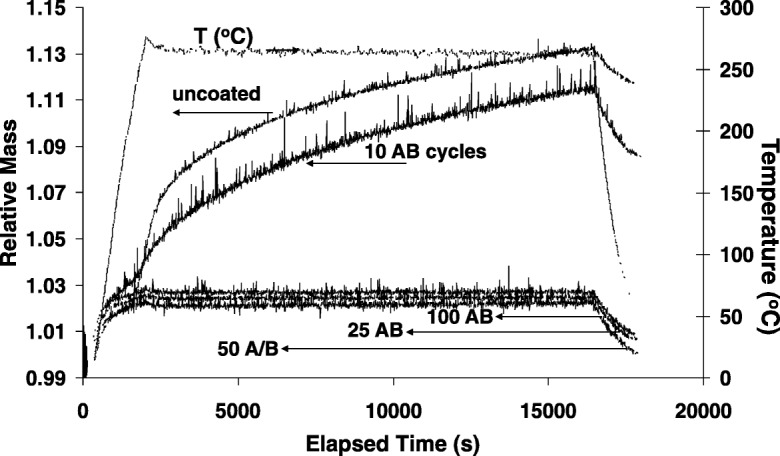
Effect of Al_2_O_3_ nanolayer on oxidation resistance of 5 μm iron powder. TGA oxidation of uncoated, 10, 25, 50, and 100 ALD AB cycles (as identified) (Wank et al. 2004a)

**Fig. 5:**
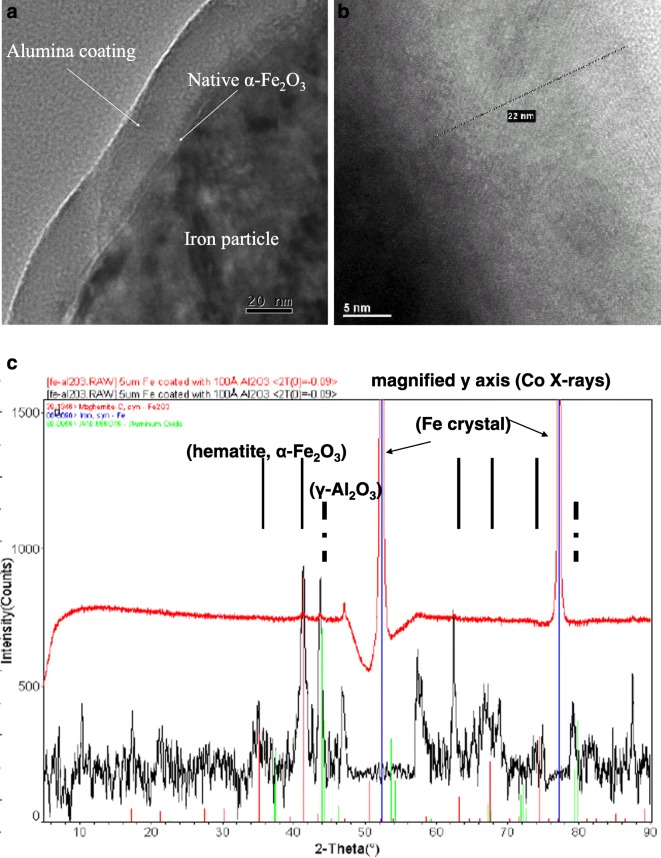
ALD alumina films on iron particles: (**a**) after 175 AB cycles, ALD Al_2_O_3_ coating on native α-Fe_2_O_3_-oxidized particle surface; (**b**) Al_2_O_3_ coating is crystalline and appears to grow epitaxially to the Fe particles; (**c**) XRD of ALD Al_2_O_3_-coated Fe showing crystalline Al_2_O_3_ film (Wank et al. 2004a)

Wank et al. (2004a) showed that 10 TMA/H_2_O cycles on 5-μm iron (Fe) particles behaved almost the same as no coating at all when these particles were oxidized in air using a TGA at 265 °C. It was not until 25 cycles that the particles were oxidation-resistant (most likely due to a uniform and continuous film coating), and 25, 50, and 100 cycles gave almost identical results (Fig. 4). Likewise, reported ICP-OES results indicated 380 mg Al/kg Fe for 5 cycles, 500 mg Al/kg Fe for 10 cycles, and 1700 mg Al/kg Fe for 25 cycles. So, broadly speaking, a substantial amount of the iron particle surface was left exposed and not coated with alumina for at least up to 10 ALD cycles. The coating was non-uniform. A uniform alumina coating occurred for some number of cycles between 10 and 25 cycles. The Al_2_O_3_ grew as a microcrystalline film using the iron crystal structure as a template for growth (Fig. 5). This aspect is nicely described by Puurunen (2005): “The ALD process modifies the chemical composition of the surface through materials deposition. The first ALD reaction cycle occurs on the surface of the original substrate material, the following cycles are usually on a surface with both the original substrate and the ALD-grown material exposed, and after several ALD reaction cycles—the exact number depending on the GPC (growth per cycle) growth (Puurunen 2003) and the growth mode—finally on a surface with only the ALD grown material exposed. If the chemical composition of the surface changes, the GPC could be expected to vary with the number of cycles.” Clearly, the particle properties are substantially different for when the substrate particle surface is still exposed to when the particle is completely encapsulated (after a number of ALD cycles), i.e., the difference between 10 and 25 cycles for the 5-μm Fe particles (Fig. 4). For the GPC of the Al_2_O_3_/Fe system, this corresponds to a film deposited with 10 ALD cycles where oxidation occurred and with 25 cycles where the particle surfaces were passivated. However, it is possible that a passivating EBC layer might have been deposited with fewer cycles than 25, but this was not investigated. According to Puurunen, it seems as if the surface of the substrate was still exposed at 10 cycles, but not at 25 cycles. Alumina typically grows at < 2 Å GPC, and so, the 10-cycle ALD process is for a film < 2 nm thick as compared to the 25 cycles where the film is > 2 nm thick. Clearly, the coating process is initially non-uniform, and the film is non-uniform until the chemical composition of the coated surface is uniform.

Although an early focus for ALD on particles was for complete pinhole-free passivating films, research was also directed towards providing ALD films without complete surface coverage in order to best maintain substrate properties. For example, boron nitride (BN) has a high thermal conductivity and its addition to composite materials is important for enhanced thermal management applications. In particular, the miniaturization of microelectronic devices has led to larger heat dissipation that requires higher thermal conductivity packaging materials. One impediment to the addition of BN particles in composites is the inertness of the BN surface basal planes. The unreactive BN surface limits the coupling between the BN particles and the epoxy matrix and lowers the BN particle loading. Hence, ultrathin films, or partial films (i.e., non-uniform, semi-continuous films), are needed to alter the chemical activity of the BN surface without significantly degrading the thermal conductivity of the BN particles.

**Fig. 6:**
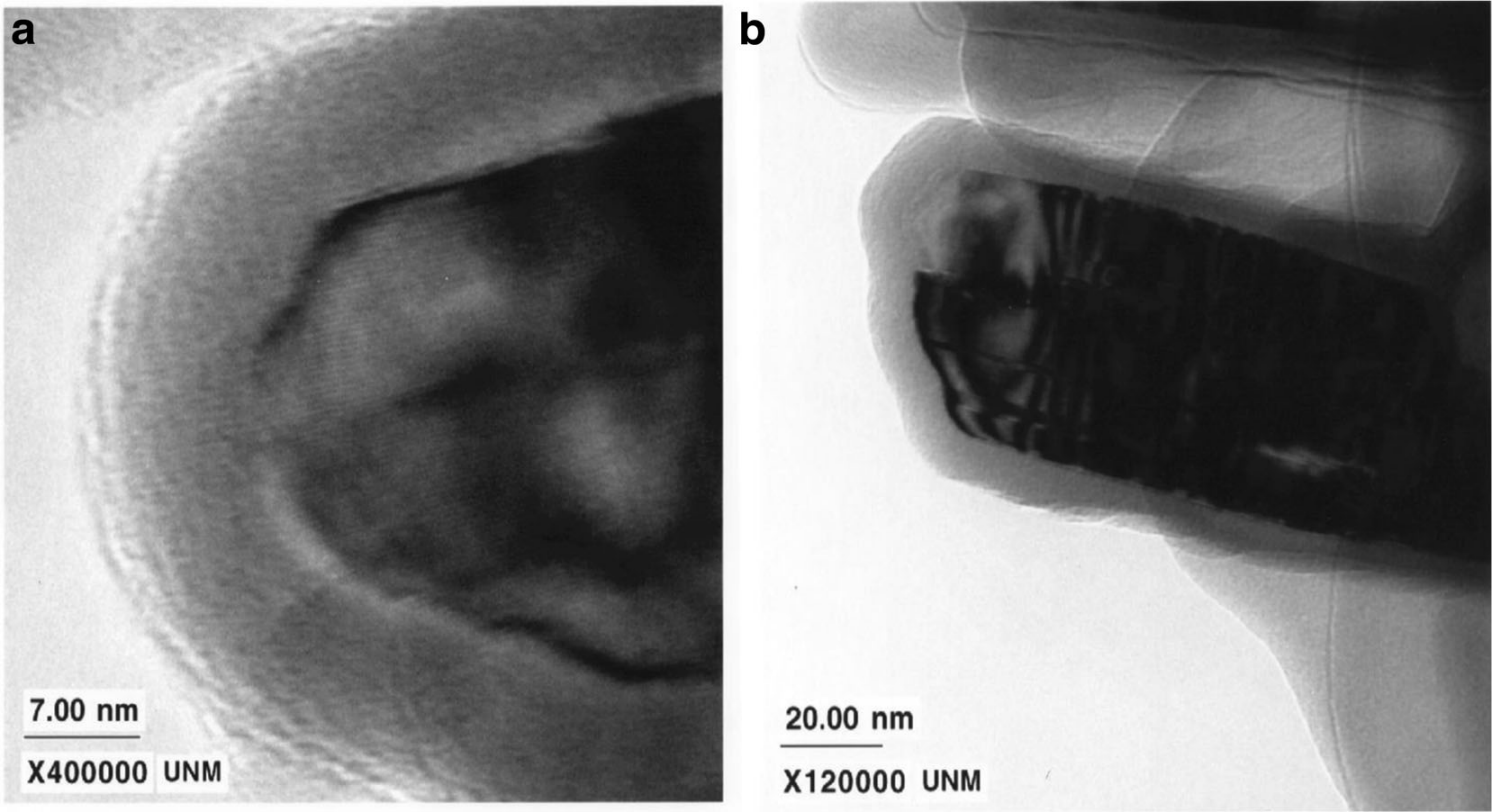
**a** Transmission electron microscope image of a BN particle coated with a 90 Å Al_2_O_3_ film after 50 AB cycles at 177 °C. The Al_2_O_3_ film is amorphous, and the crystalline graphitic planes are visible in the BN particle. **b** Transmission electron microscope image of a BN particle coated with a 90 Å Al_2_O_3_ film after 50 AB cycles at 177 °C. The Al_2_O_3_ film is deposited equally well on the basal planes and edges of the BN particles (Ferguson et al. 2000b)

**Fig. 7:**
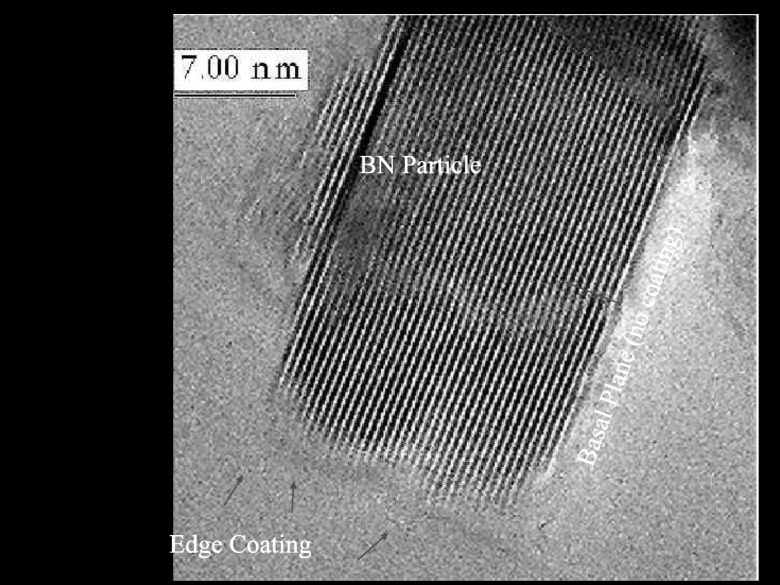
HRTEM of silica ALD film on boron nitride particle showing edges coated, but basal planes with patchy partially covered film (Ferguson et al. 2000a)

**Fig. 8:**
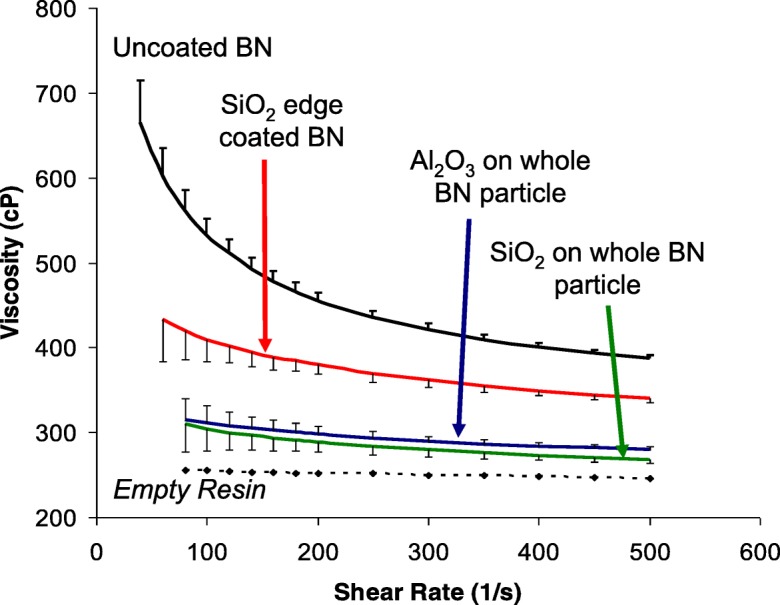
Effect of coating morphology on viscosity (10% filler volume; BN is trade name PT120, sold by Advanced Ceramics, Inc.)

Coupling agents have been developed for oxide particle surfaces and the epoxy matrix. Oxide films deposited on the BN particles could utilize these same coupling agents. To minimize the effect of the oxide coating on the thermal conductivity of the BN particles, the oxide film should be ultrathin, or, even incomplete. An increased loading of BN particles in the epoxy resin increases viscosity to the point where flowability negatively impacts the application. The optimal system is one where the functionalized BN particles can have increased loading for improved epoxy thermal conductivity, but without negatively impacting viscosity and, hence, flow ability of the filled resin. It was shown that an alumina ALD film of only 2 nm thick would decrease thermal conductivity by 50% (Wank et al. 2004b); hence, the research direction was for ultrathin films of only 1 nm or less, or a non-uniform, semi-continuous partial surface coverage in order to maintain high TC while improving coupling with the resin matrix. Ferguson et al. (2000a, b) showed that the BN edge planes were covered with reactive BOH* and BNH_2_* surface species, while the BN basal planes do not have these chemical species to assist in nucleation but instead have an electron lone pair residing on the nitrogen atom. They further showed that TMA is a Lewis acid that can readily accept an electron pair, and so, this Lewis acid-base interaction facilitates the deposition of alumina on the basal planes of the BN particles, encapsulating the entire particle (Fig. 6). However, it has been shown that longer exposures to TMA are required to coat the basal planes relative to the edges (Wank et al. 2004b). On the other hand, silicon tetrachloride (SiCl_4_) used as an ALD precursor for silica does not have this functionality and, hence, preferentially coats the active edges of the BN particle, leaving the basal planes uncoated or having patchy films (Ferguson et al. 2000a, b) (Fig. 7). The effect of complete (BN/Al_2_O_3_, BN/SiO_2_-Al_2_O_3_) and incomplete (BN/SiO_2_) coatings on 10% filled resin viscosity is shown in Fig. 8.

## Agitated particle bed reactors

**Fig. 9:**
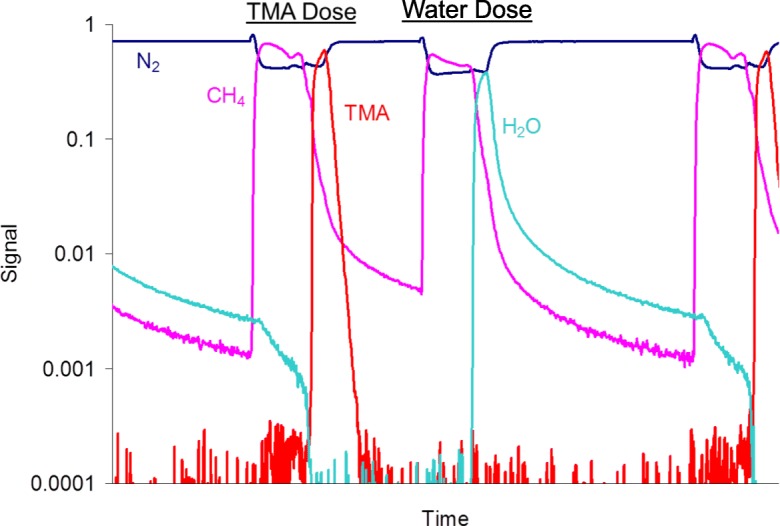
Downstream mass spectrometer signal for 1 cycle of Al_2_O_3_ particle ALD, indicating highly efficient precursor use. TMA dose: 2AlOH* + 2Al(CH_3_)^3^ → 2[Al-O-Al(CH_3_)^2^]* + 2CH_4_. H2O dose: 2[Al-O-Al(CH_3_)^2^]* + 3H_2_O → Al_2_O_3_ + 2AlOH* + 4CH_4_. Overall: 2Al(CH_3_)^3^ + 3H_2_O → Al_2_O_3_ + 6CH_4_

**Fig. 10:**
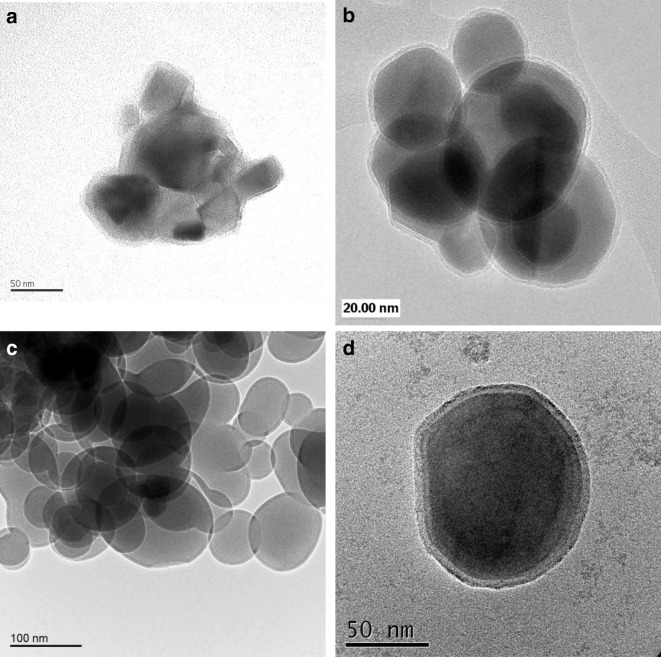
Particle ALD for fixed bed (**a**, **b**) and agitated bed (**c**, **d**) processing: (**a**) Li_4_Ti_5_O_12_ particles coated with a TiN film by ALD (Snyder et al. 2007); (**b**) ZrO2 particles coated with a BN film by ALD (Ferguson et al. 2002); (**c**) SiO_2_ particles coated with an Al_2_O_3_ film by ALD (Hakim et al. 2007a); and (**d**) TiO2 nanoparticles coated with a SiO2 film by ALD (Liang et al. 2010)

Agitated particle bed reactors, such as fluidized bed (Wank et al. 2004b; Hakim et al. 2005a, b; King et al. 2007) and rotary reactors (McCormick et al. 2007a, b), have been developed for carrying out particle ALD (Liang et al. 2009b). Other configurations have been proposed and tested as well, but the original engineering scale-up was with fluidized beds. Wank et al. (2004b) were the first to show that primary particles are coated by ALD using fluidized bed coating reactors. A mass spectrometer (M/S) can be used downstream of the fluidized bed in order to follow the progress of the ALD chemistry surface reactions (King et al. 2007). A unique characteristic of particle ALD in fluidized bed reactors is that the coating process can be carried out with almost 100% use of precursor chemicals, depending on the ALD chemistry. It can be seen in the M/S trace in Fig. 9 that breakthrough of expensive TMA precursor does not occur until virtually all of the active sites are converted as indicated by a drop-off in CH_4_, the byproduct of the A reaction. The same occurs for H_2_O breakthrough for the B reaction. This is the result of self-limiting surface reactions. Within the fluidized bed processing, precursors seek unreacted sites. Reaction occurs almost instantaneously with TMA until all hydroxyl sites are converted to methyl sites. Then, the breakthrough of the unreacted TMA results. The same occurs for the B reaction. The net effect is that near 100% of the specific precursor can be reacted and not wasted by bypassing out of the system (Grillo et al. 2015). If one knows how much surface area is in the bed to be coated and the precursor feed rate, one can even carry out process control to stop dosing in order to potentially prevent any purge from being required. The particle ALD processing is independent of mixing time of the substrate in the bed relative to the location of the precursor feed inlet. This is not true for CVD in a fluidized bed where over-coating could occur if the mixing time were not fast enough compared to the feed rate of the precursor; there would be a distribution in film thickness. For particle ALD in agitated systems, all particles are identically coated because of the self-limiting surface chemistry. Fixed-bed processing of particle ALD can lead to some non-uniform coating and bridging of substrate particles (see Fig. 10a,b) which does not occur for agitated processing (Fig. 10c, d). This aspect of particle ALD in agitated systems (fast reactions that are self-limiting), in which the precursor waste is minimized/eliminated, is what contributes to making particle ALD a low-cost process. Although ALD generally proceeds with film growth at 1 or 2 Å/cycle, rapid silica deposition (Hausmann et al. 2002) has been demonstrated on large quantities of cohesive nanoparticles using a fluidized bed reactor (Liang et al. 2010) (Fig. 10d). A growth rate of ~ 1.8 nm/cycle was achieved for an underdosed and incomplete polymerization reaction on 18 and 160 nm TiO_2_ particles.

## Coating primary nanoparticles

**Fig. 11:**
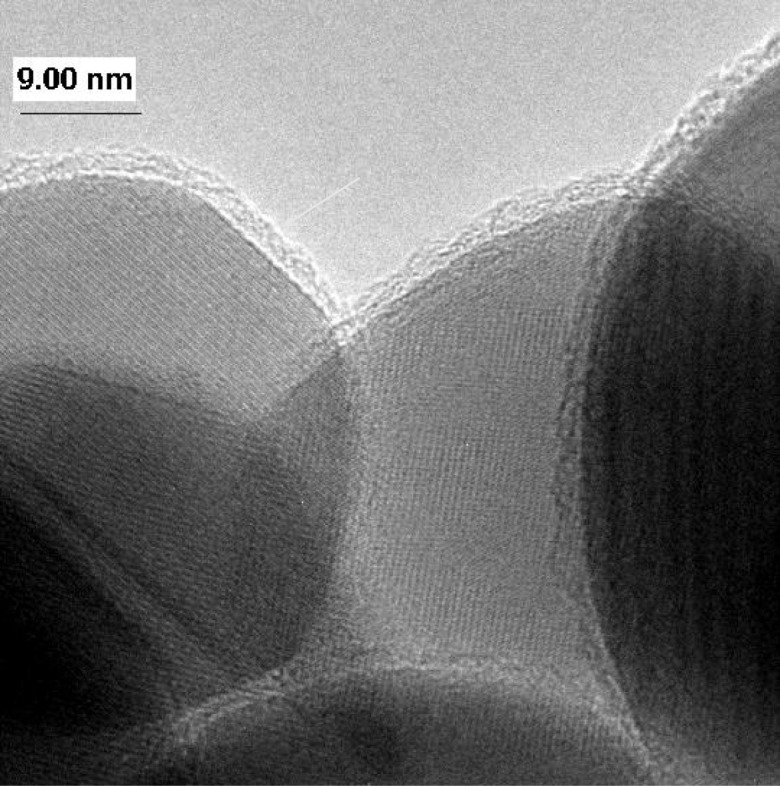
Boron nitride ALD nanofilm (2.5 nm) grown on a ZrO_2_ nanoparticle (26 ALD cycles at 227 °C) (Ferguson et al. 2002)

**Fig. 12:**
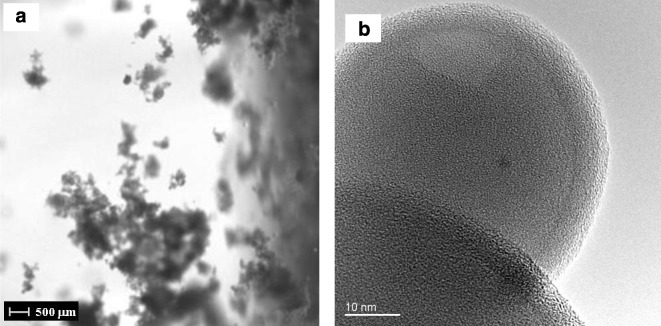
Nanocoating primary silica particles by ALD in a fluidized bed reactor: (**a**) fluidized aggregates of Aerosil OX-50 fumed silica nanoparticles and (**b**) TEM image of alumina-coated silica nanoparticles after 50 ALD TMA/H_2_O coating cycles (5.2 nm film thickness) (Hakim et al. 2005a)

Ferguson et al. (2002), using a fixed-grid FTIR system, demonstrated coating ~ 50 nm zirconia (ZrO_2_) nanoparticles (20.2 m^2^/g) with conformal ~ 2.5 nm films of boron nitride (BN) after 26 AB cycles of boron trichloride (BCl_3_) and ammonia (NH_3_) precursors at 227 °C. High-resolution transmission electron micrographs (HRTEMs) revealed uniform and conformal BN films (see Fig. 11). Hakim et al. used a fluidized bed reactor and demonstrated that large quantities of primary silica, SiO_2_ (~ 40 nm) (Hakim et al. 2005a), and ZrO_2_ (~ 26 nm) (Hakim et al. 2005b) nanoparticles are coated individually (Fig. 12b) and conformally with Al_2_O_3_ by ALD despite their high aggregation tendency during fluidization (Fig. 12a). For the coated ZrO_2_, transmission Fourier transform infrared (FTIR) spectroscopy was performed ex situ. Bulk Al_2_O_3_ vibrational modes were observed for coated particles after 50 and 70 cycles, and no features of the ZrO_2_ spectrum appeared. Coated nanoparticles were also examined with transmission electron microscopy (TEM), high-resolution field-emission scanning electron microscopy (FESEM), energy-dispersive spectroscopy (EDS), and X-ray photoelectron spectroscopy (XPS). Analysis revealed highly conformal and uniform alumina nanofilms throughout the surface of the nanoparticles. For SiO_2_ nanoparticle coating, the highest intensity peaks for SiO_2_ and Si were completely attenuated, as expected for a conformal film. The particle size distribution (PSD) of the nanoparticles was not affected by the coating process, thus, indicating that agglomeration did not occur. Further, the specific surface area of the nanoparticles varied slightly with particle ALD as theoretically expected for the measured film thickness. This detailed analytical characterization of particles coated by ALD in a fluidized bed provided validation that all primary particles were coated uniformly with pinhole-free films.

Even though the nanoparticles were fluidized with larger aggregates (Fig. 12a), they were individually coated with conformal films (Fig. 12b). The particles were not agglomerated. Part of the explanation is proposed by Hakim et al. (2005c) who investigated the fluidization of nanopowders using a high-speed laser imaging system in real time. Although fluidization of aggregates is dictated by interparticle forces, they found that fluidized aggregates show a dynamic behavior where outer edges are shed and picked up by other aggregates. The relatively large size of aggregates of nanoparticles and their frequent collisions with other large aggregates while in continuous flow promote this dynamic behavior. So, during fluidization, aggregates of nanoparticles continuously break apart and form. The aggregates do not maintain a stagnant size or shape. This “dynamic equilibrium,” or, dynamic aggregation, between inertial and cohesive forces is a unique characteristic of fluidized nanoparticles. In this manner, all particle surfaces are exposed to the surrounding gas and ALD can deposit conformal films on the agitated primary nanoparticles.

## Molecular layer deposition

**Fig. 13:**
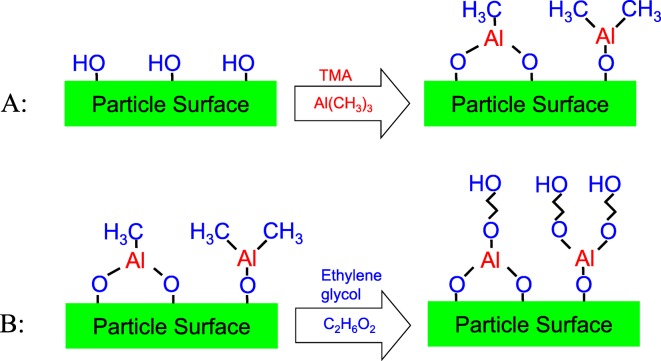
Half-reactions of aluminum alkoxide (alucone) MLD. A: –OH* + Al(CH_3_)^3^ → –OAl(CH_3_)^2^* + CH_4_. B: –AlCH_3_* + OH(CH_2_)2OH → –AlO(CH_2_)^2^OH* + CH_4_

Many techniques, such as the chemical vapor deposition (CVD) method (Kubono et al. 1991) and the Langmuir-Blodgett method (Sanchez-Gonzalez et al. 2003) have been developed for fabricating polymeric thin films. However, these methods cannot control the order and location of molecular compounds on the substrate surfaces, which may inhibit the desired functionality of the coatings. The molecular layer deposition (MLD) method (Yoshimura et al. 1991; Yoshimura et al. 1992), which is similar to ALD and is also based on sequential, self-limiting surface reactions, can be utilized to deposit polymer films on particles. While ALD enables material growth at the atomic level, MLD controls growth at the molecular level. In this process, molecules are stacked on the substrates one by one in order of preference. Hence, the MLD GPC rate for producing polymers is higher than the corresponding atomistic GPC rate for ALD. The MLD technique offers the same advantages for polymer film deposition as ALD does for ceramic films. In addition, MLD can deposit hybrid polymer films using suitable precursors, such as trimethylaluminum (TMA) and ethylene glycol (EG) for aluminum alkoxide (alucone) hybrid polymer(Dameron et al. 2008) (Fig. 13). This vapor-phase method, which operates in vacuo and does not require solvents or catalysts, is a useful and promising technique for the fabrication of functional ultrathin polymeric layers (Meng 2017a, b).

**Fig. 14:**
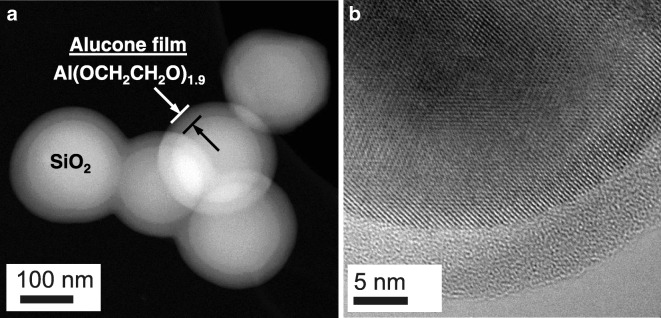
MLD films observed by STEM and TEM: (**a**) 50 cycles MLD-coated 250 nm SiO_2_ particles, ~ 25 nm alucone film, 0.5 nm/cycle at 100 °C; (**b**) 20 cycles MLD-coated 160 nm TiO_2_ particles, ~ 7 nm alucone film, 0.35 nm/cycle at 160 °C (Liang et al. 2009a)

Ultrathin aluminum alkoxide (alucone) polymer films were deposited on primary silica and titania nanoparticles using molecular layer deposition (MLD) in a fluidized bed reactor from 100 to 160 °C (Liang et al. 2009a). Similarly, zinc alkoxide films have been deposited as well (Liang et al. 2012a). In situ mass spectrometry revealed that the growth of alucone MLD films was self-limiting as a function of the individual trimethylaluminum and ethylene glycol exposures. The composition and highly conformal alucone films throughout the surface of both silica and titania nanoparticles were confirmed. The highest growth rate was observed at the lowest sample temperature. Primary nanoparticles were coated individually despite their strong tendency to aggregate during fluidization. The composition of alucone hybrid nanolayers was confirmed by EDS. Highly conformal and uniform films on silica and titania particles were observed via z-contrast STEM (Fig. 14a) and TEM (Fig. 14b), respectively. The thickness and molecular weight of polymeric thin films can be effectively controlled by the number of reaction cycles during the sequential vapor deposition process.

### Ultrathin microporous/mesoporous metal oxide films

**Fig. 15:**
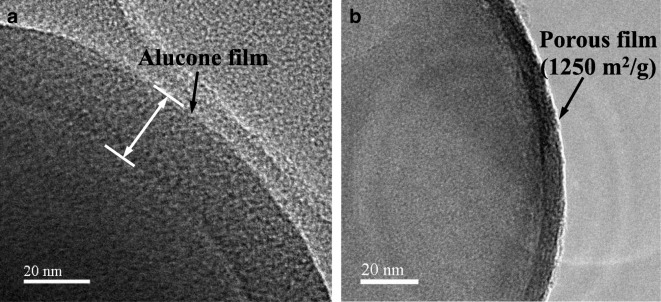
TEM images of porous films formed by oxidation in air: (**a**) ~ 25 nm-thick alucone MLD as deposited; (b) ~ 8 nm-thick porous alumina film after oxidation in air at 400 °C, 1250 m^2^/g (Liang et al. 2009b)

Porous aluminum oxide films with precisely controlled thickness down to several angstroms are formed on particle surfaces upon calcination or water etching of dense aluminum alkoxide hybrid polymer films deposited by MLD (Liang et al. 2009b, 2013). The organic constituents of the MLD films are removed by etching or calcination. Because of the layer-by-layer growth process for MLD, the deposited polymer films have regular structure, and the oxidation of the MLD polymer films produces uniform interconnected highly porous structures with high surface area. The porous structure has both micropores and mesopores. Since carbon is removed during the calcination process in air, the length of the carbon chains in the hybrid organic/inorganic MLD polymer films helps determine the size of the pores in the alumina film. In an ideal case, a monolayer-by-monolayer MLD process occurs and the length of the carbon chains in the hybrid polymer films determines pore size. So, porous oxide films obtained from organic precursors with longer carbon have larger pores. An alucone polymer film deposited on spherical silica particle surfaces based on a 3-step ABC reaction sequence (Liang et al. 2013) using TMA, ethanolamine (EA), and maleic anhydride (M)A as the reactants resulted in larger pores than those formed using a 2-step AB MLD process. This method has great potential to selectively tune the size of alumina nanopores by oxidizing alucone thin films having different polymer compositions. The surface area of the resulting porous films from AB MLD has been reported to be between ~ 330 and 1250 m^2^/g following calcination at 1000 and 400 °C, respectively. Micropore diameter varies between about 0.6 and 0.8 nm depending on the AB or ABC MLD synthesis chemistry. HRTEMs are shown for the MLD-coated particles (Fig. 15a) and the resulting porous alumina film (Fig. 15b) on 250 nm spherical silica particles before and after air calcination at 400 °C. The film thickness decreased from 25 to 8 nm as the organic constituent was removed. This porous thin film/coating growth technique will potentially have wide applications in catalyst functionalization, controlled drug delivery, and nanofiltration.

## Coating polymer particles

Improving polymer properties can benefit the multitude of uses for polymers. The high gas permeability of polymers is one property that limits their use in various food, medical, and electronic packaging applications (Chatham 1996; Erlat et al. 1999; Weaver et al. 2002). Inorganic materials typically have a much lower gas permeability than polymers. When used as coatings on polymers, these inorganic materials can serve as gas diffusion barriers and can dramatically improve the polymer performance (Chatham 1996; Erlat et al. 1999; Weaver et al. 2002). However, polymers are thermally fragile. Low-temperature deposition techniques, such as sputtering, evaporation, and plasma-enhanced chemical vapor deposition (CVD), have been required to deposit the inorganic diffusion barrier (Erlat et al. 1999). Because inorganic materials are brittle, thin inorganic diffusion barriers on polymers are needed to maintain polymer flexibility without cracking. The optimum thickness for maximum flexibility is as thin as possible, but thick enough to provide the specific barrier performance. For these small thicknesses, line-of-sight deposition techniques, such as sputtering and evaporation, are limited by defects and pinholes. The continuous and pinhole-free ALD film characteristics are important for gas-diffusion barriers.

**Fig. 16:**
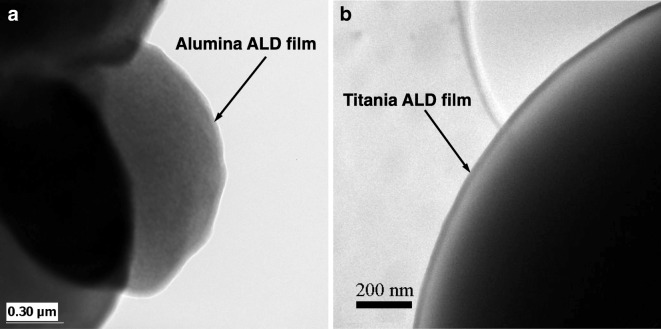
ALD films deposited on polymers: (**a**) alumina deposited by ALD on 3 μm HDPE at 77 °C (Ferguson et al. 2004); (**b**) titania deposited with 200 cycles of titanium tetraisopropoxide (TTIP)/H_2_O_2_-coated HDPE particles (16 μm in diameter) at 80 °C (Liang et al. 2009b)

The reactions between TMA and various polymer substrates were studied using in situ Fourier transform infrared (FTIR) spectroscopy (Gong and Parsons 2012). It was found that TMA reacts with certain nucleophilic functional groups on the polymer surface during the initial ALD cycles. TMA initially binds to Lewis basic atoms of common organic functional groups OH, NH_2_, and NO_2_ (Yang et al. 2017). For some polymers, TMA penetrates into the polymer and reacts in the bulk. Ferguson et al. (2004) and Liang et al. (2007b) carried out sequential exposures of TMA and H_2_O at 77 °C to encapsulate low-density polyethylene (LDPE) particles with an ultrathin Al_2_O_3_ film. FTIR studies revealed that the nucleation of Al_2_O_3_ atomic layer deposition (ALD) on the LDPE particles occurred primarily via adsorption of TMA onto the LDPE surface or absorption of TMA into the LDPE particle followed by the reaction with H_2_O. The FTIR spectra then revealed the progressive switching between AlCH_3_* and AlOH* species with alternating exposure to TMA and H_2_O. This nucleation of Al_2_O_3_ ALD did not require the existence of specific chemical functional groups on the polymer. The FTIR spectra also demonstrated that the sequential exposures of TMA and H_2_O led to an increase in Al_2_O_3_ bulk vibrational modes. The increase of the absorbance for the Al_2_O_3_ bulk vibrational modes was linear with the number of AB cycles. The presence of an Al_2_O_3_ film on the LDPE particles was confirmed using transmission electron microscopy (TEM), FTIR, and XPS (Ferguson et al. 2004; Liang et al. 2007b) (Fig. 16a).

**Fig. 17:**
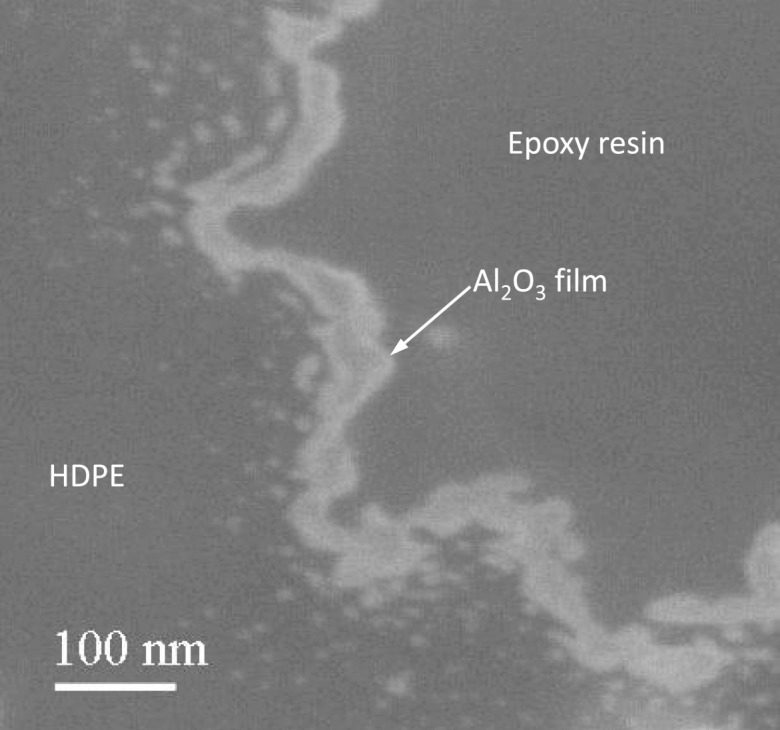
Focused ion-beam cross-sectional scanning electron micrograph (FIB-SEM image) of Al_2_O_3_-coated high-density polyethylene (HDPE) particle (60 μm) after 100 ALD cycles (Liang et al. 2007b)

Similar coatings were carried out using TTIP and H_2_O_2_ on 16 μm HDPE at 80 °C (Fig. 16b). The TEM images along with particle size distribution and surface area analysis revealed that the Al_2_O_3_ and TiO_2_ coatings were very conformal to the LDPE and HDPE particles and that the polymer particles did not aggregate. The Al_2_O_3_ coating was also thicker than expected from typical Al_2_O_3_ ALD growth rates. This thicker Al_2_O_3_ coating was explained by the presence of hydrogen-bonded H_2_O on the Al_2_O_3_ surface that increases the Al_2_O_3_ growth rate during TMA exposures. The ALD film grew on the particle surface without functional groups to react. Investigation using focused ion beam (FIB) cross-sectional SEM imaging allowed precise observation at the edge interface of the polymer and Al_2_O_3_ film. The FIB cross-sectional SEM image of the HDPE particles (60 mm) after 100 cycles is shown in Fig. 17. Al_2_O_3_ islands began to grow below the polymer surface, and the film merged into a linear layer as it grew. Approximately 40 nm-thick Al_2_O_3_ films were coated on the polymer surface. This thickness represents a growth rate of about 0.4 nm per coating cycle at this experimental condition. The SEM image (Fig. 17) also shows that the Al_2_O_3_ films appear to be very uniform and smooth.

**Fig. 18:**
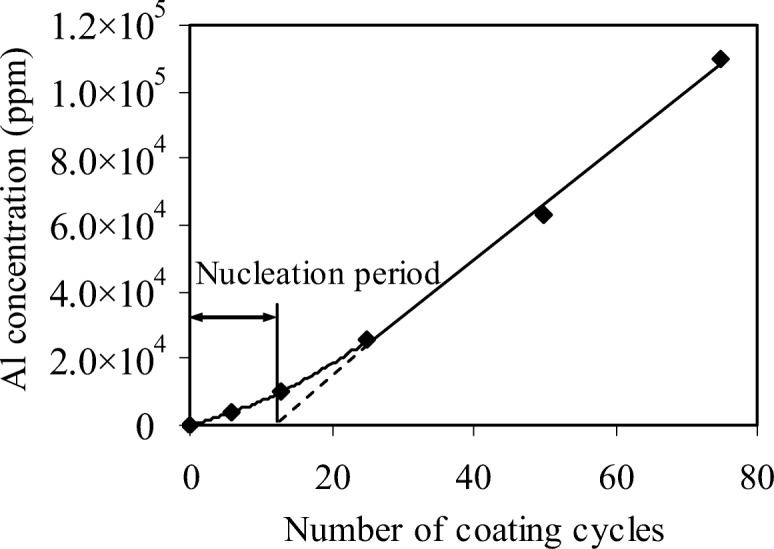
Aluminum concentration on high-density polyethylene (HDPE) particles versus number of ALD coating cycles (Liang et al. 2007b)

**Fig. 19:**
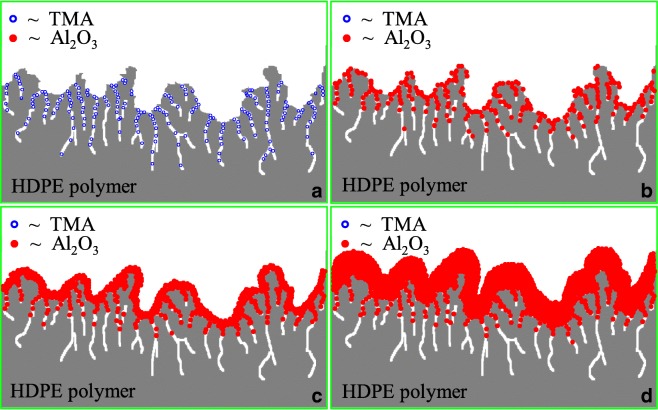
Proposed Al_2_O_3_ growth mechanism (Liang et al. 2007b)

The Al_2_O_3_ ALD is conventionally thought to begin with native hydroxyl groups on the surface. HDPE, however, is one kind of saturated hydrocarbon which lacks typical chemical functional groups, such as hydroxyl species, that are necessary to initiate the growth of an inorganic film. So, the fundamental concept of Al_2_O_3_ ALD cannot take place on the HDPE particle surface. The nucleation of Al_2_O_3_ ALD on HDPE requires a mechanism that does not involve the direct reaction between TMA and HDPE. Consequently, an alternative mechanism is needed to explain the Al_2_O_3_ ALD on HDPE. HDPE has a porous surface, which is due to the interstitial space between individual molecules as HDPE does not have the regular lattice-type structure found in metals. Both HDPE and TMA are non-polar; so, it is expected that TMA has a reasonable solubility in the HDPE particles and that TMA can adsorb onto the surface of the polymer and subsequently diffuse into the near-surface regions of the polymer. During the ALD reaction, TMA will be first exposed to the HDPE particles and diffuse into the bulk of the polymer matrix; therefore, the incoming H_2_O will react efficiently with TMA molecules at or near the surface of the polymer particles and Al_2_O_3_ clusters will be formed. The pores on the particle surface will become smaller and will gradually close with progressive coating cycles. After several coating cycles, the Al_2_O_3_ clusters will eventually merge to create a continuous adhesion layer on the polymer particle surface. This phenomenon can be observed in Fig. 17. Al_2_O_3_ clusters with hydroxyl groups will provide a “foothold” for the deposition of Al_2_O_3_ films on the polymer. As shown in Fig. 18, the concentration of aluminum is almost directly proportional to the number of coating cycles after 25 cycles, which indicates a constant growth rate and a linear dependence between the film thickness and number of growth cycles after a nucleation period. The mechanism of ALD nucleation on polymers may be dependent on a number of factors, including chemical functional groups on or in the polymer, diffusion and solubility of the ALD reactants in the polymer, and the polymer structure and temperature. The model of the predicted growth mechanism is illustrated in Fig. 19. However, polyester, polymide, and polyether are more reactive, and in situ FTIR spectra showed a larger extent of reaction with TMA, facilitating the nucleation of an ALD film on these polymers (Gong and Parsons 2012).

### Porous polymer/ceramic composite materials and use as templates

**Fig. 20:**
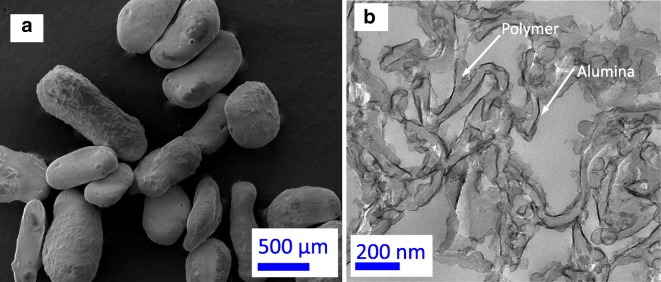
Conformal Al_2_O_3_ films coated on porous poly(styrene-divinylbenzene) particles: (**a**) FESEM images of porous PS-DVB particles; (**b**) cross-sectional TEM image of Al_2_O_3_-coated porous polymer particles after 25 ALD cycles (Liang et al. 2007a)

A fluidized bed reactor was used to deposit alumina films by ALD on internal and external highly porous (~ 85% porosity, 8–10 cm^3^/g pore volume, 43.5 m^2^/g surface area, 70 kg/m^3^ particle density, and ~ 600 μm diameter) co-polymerized polystyrene (styrene-divinylbenzene, PS-DVB) particle surfaces (Fig. 20a) at 33 °C under low-pressure conditions (~ 3 Torr) (Liang et al. 2007a). The 33 °C reaction temperature was much lower than the softening/melting temperature point of the porous polymer particles. The XPS measurements revealed that alumina films were deposited on the polymer particle surfaces. The results of EDS indicated that the alumina films were deposited throughout the inner and outer surfaces of the porous particles. STEM and cross-sectional TEM investigations revealed highly conformal and uniform alumina coatings. Combined with STEM and cross-sectional TEM, the surface area and aluminum concentration based on ICP-AES versus the precursor dose time and coating cycles revealed that the pore filling mechanism of alumina ALD for this highly porous polymer was a conformal coating of the pore walls. The cross-sectional TEM image of the porous PS-DVB particles after 25 cycles is shown in Fig. 20b. The black threads in the image are alumina films, which appear to be very uniform and smooth. Obviously, on the wall of some pores, there are no alumina films, which may have been peeled off during the cutting process. On the basis of the TEM image, approximately 7 ± 2 nm-thick alumina films were coated on the wall of the pores of the polymer particle. This thickness also represents a growth rate of about 0.3 nm per coating cycle at this experimental condition, which corroborates the film thickness observed by STEM.

Porous alumina and other ceramic particles or structures with crystallized frameworks and controlled nanometer wall thickness can be easily fabricated by ALD. A sacrificial template, such as a polymer, can be coated by ALD, and then, the sacrificial substrate was removed leaving behind a unique ceramic structure. Liang et al. (2012b) demonstrated that the highly porous ALD-coated PS-DVB polymer particles (Liang et al. 2007a) could be calcined in air to remove the polymer template and leave a porous ceramic structure with precise wall thickness corresponding to the ALD growth rate and number of ALD cycles carried out. Surprisingly, for Al_2_O_3_ ALD, the structure did not collapse and mimicked the starting morphology of the polymer. A mesoporous structure of crystalline Al_2_O_3_ with a high specific surface area and large pore volume was formed for calcination temperatures above 600 °C. Porous crystalline alumina with a surface area of 80–100 m^2^/g was obtained and was thermally stable at 800 °C. Such porous alumina particles may find wide application in nanotechnology and catalysis.

### Metal ALD films and seed layers on polymer particles

Tungsten (W) ALD was investigated on a variety of polymer particles, including polyethylene (PE) MW-1100, polyvinylchloride (PVC) MW-90,000, polystyrene (PS) MW-190,000, and polymethylmethacrylate (PMMA) MW-15,000 (Wilson et al. 2008). The polymer particles were placed in a rotary ALD reactor without any prior treatment. The W ALD was performed at 80 °C using tungsten hexafluoride (WF_6_) and disilane (Si_2_H_6_) as the gas phase reactants. The nucleation of W ALD directly on the polymer particles at 80 °C required > 50 AB cycles. In contrast, the polymer particles treated with only 5 AB cycles of Al_2_O_3_ ALD to provide a “seed layer” were observed to blacken after 25 AB cycles of W ALD. XPS analysis of the W 4f peaks after W ALD on the polymer particles was consistent with a WO_3_ thickness of 29 Å covering the W ALD film. The oxidation of the W ALD film may be dependent on the radius of curvature of the polymer particles. W ALD on polymers may have applications for flexible optical mirrors, electromagnetic interference shielding, and gas diffusion barriers.

## Carbon nanotubes

**Fig. 21:**
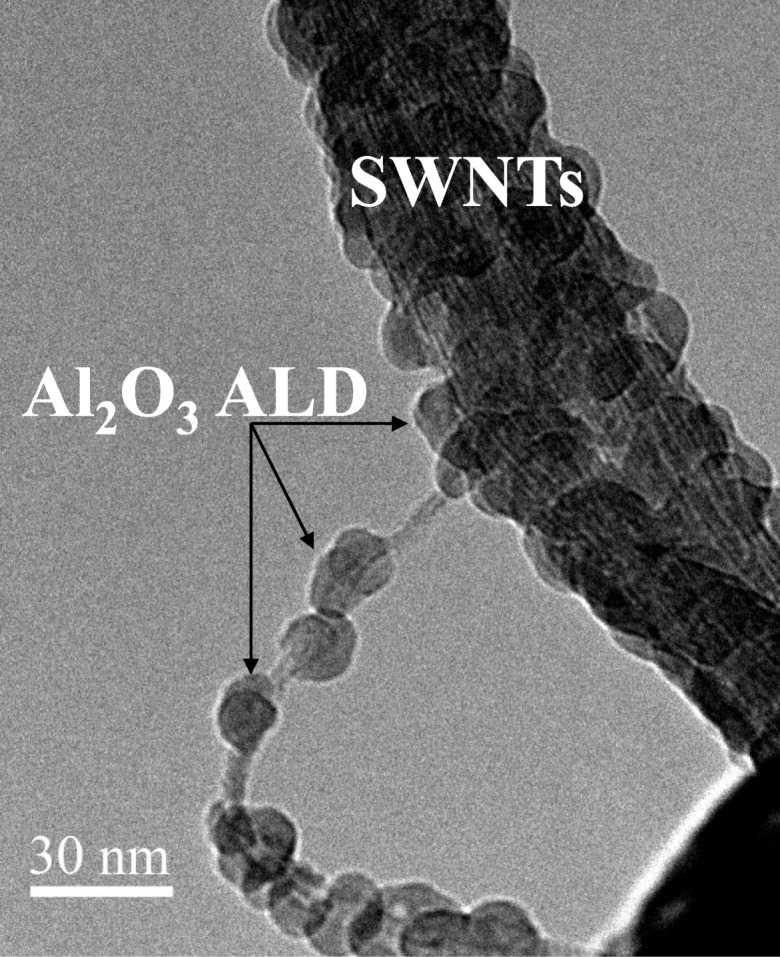
Transmission electron micrograph image (TEM) of a typical Al_2_O_3_ ALD nodule growth pattern of a non-functionalized single-walled nanotube (SWNT) bundle (Zhan et al. 2008)

**Fig. 22:**
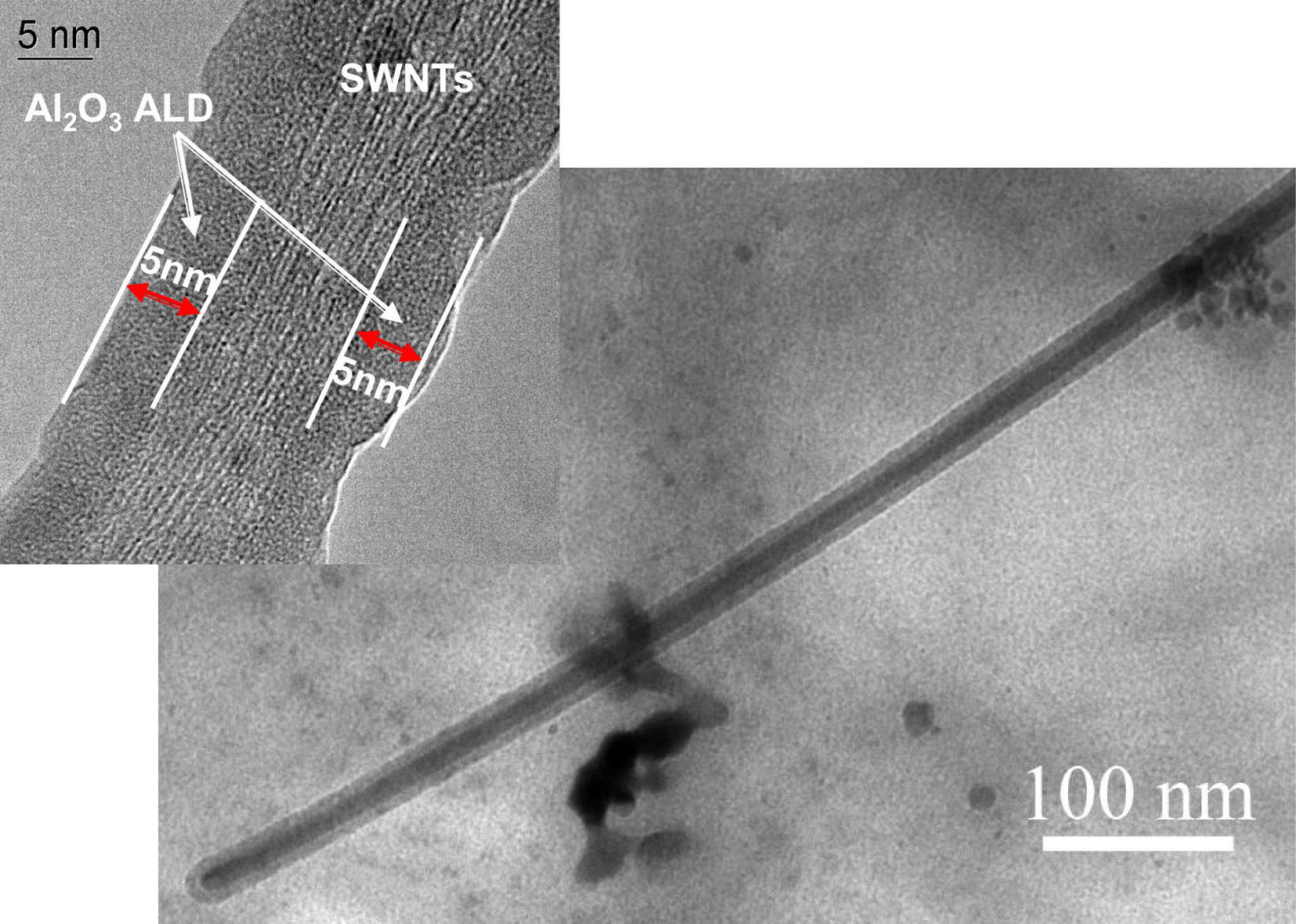
Al_2_O_3_ ALD on SDS (sodium dodecylsulfate) surfactant-dispersed SWNTs showing (**a**) a smooth, conformal film in the radial and axial directions of the nanotube bundle and (**b**) high-resolution TEM image of the same, showing radial growth from closely packed nodules that forms a continuous film (Zhan et al. 2008)

**Fig. 23:**
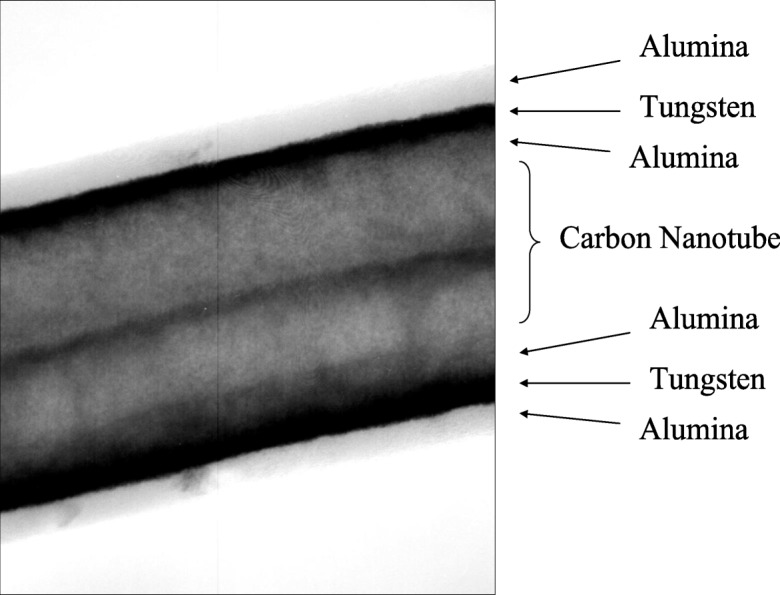
World’s smallest coaxial cable by particle ALD (Al_2_O_3_/W/Al_2_O_3_), bilayer on carbon nanotube (Herrmann et al. 2005)

There is a technological need to develop a scale-able process for functional thin-film deposition on bulk-grown non-covalently functionalized carbon nanotubes for integration into a broad spectrum of devices for a variety of applications from back-end interconnects to biological probes. Nanotubes possess many extraordinary properties, such as high strength and large electrical conductivity. The success of many of these applications depends on the deposition of insulating, passivating, or functional films on the carbon nanotubes. ALD was carried out on high-aspect ratio single-walled (SWCNTs) (Zhan et al. 2008) and on large quantities of multi-walled (MWCNTs) (Cavanagh et al. 2009) carbon nanotubes using fluidized bed and rotary reactors, respectively. Other studies used fiber baskets for safe containment of CNTs in conventional viscous flow reactor systems, thus, circumventing the use of fluidized bed and rotary reactors (Devine et al. 2011). Conformal and uniform deposition of ultrathin insulating films resulted for ALD on liquid-phase-pretreated SWNTs. Dispersion techniques served the dual role of separating and functionalizing SWNTs for ALD precursor nucleation, relative to control samples in the as-received state or dispersed in H_2_O alone. Isolated nodule growth was attainable on non-functionalized control samples (Fig. 21), but it resulted in a significant degree of exposed, non-passivated SWNT surface area. The surface density of Al_2_O_3_ nodules on ethanol-treated SWNTs was significantly greater than that of the control, and continuous films were achieved within a reasonable number of coating cycles. Conformal Al_2_O_3_ films were only achievable using ethanol and sodium dodecylsulfate (SDS)-based surfactant dispersion techniques (Fig. 22). XPS confirmed the presence of sulfur after ALD coatings were deposited on SDS-treated SWNTs, which supports the notion that the physisorbed micellar structure promoted the conformal ALD growth. Atomic layer-deposited Al_2_O_3_ was grown at a rate of 0.13 nm/cycle at 177 °C. Bundle sizes were significantly reduced using these dispersion techniques, as verified by electron microscopy, relative to control samples. Insulating, multilayered, and functionalized ALD coatings were deposited conformally on multi-walled carbon nanotubes by ALD (Fig. 23) (Herrmann et al. 2005). Multilayered coatings consisted of alternating layers of dielectric and conductive materials, Al_2_O_3_ and W, respectively. This coated carbon nanotube can function as a nanoscale coaxial cable.

## Functionalized particulate materials

**Fig. 24:**
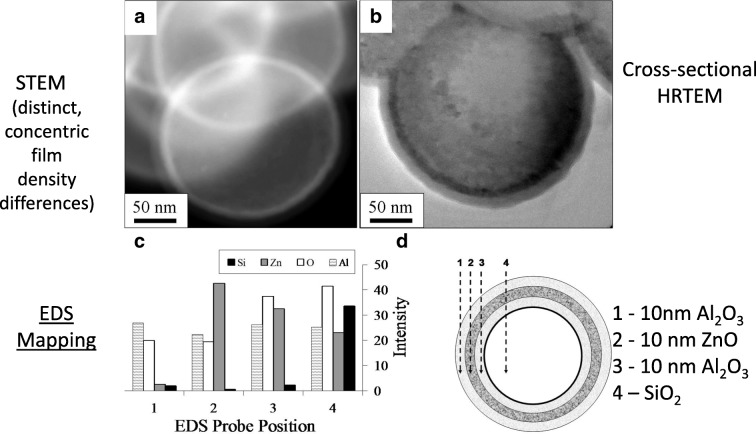
Stable, conformal multilayered concentric shells of Al_2_O_3_ on ZnO on Al_2_O_3_ on spherical SiO_2_ nanoparticles deposited via ALD; (**a**) STEM image of coated particles; (**b**) TEM image of cross-sectioned particles; (**c**) elemental EDS mapping across the four locations depicted in the (**d**) schematic of the multilayered film (King et al. 2009a)

Since particle ALD can be applied to primary particles at the atomic scale, it provides a surface modification to individual particles while maintaining bulk properties. Multi-layered films can be produced (Fig. 24). As discussed previously, the first applications of particle ALD were (1) use as a barrier film to passivate micron-sized iron particle surfaces from oxidation (Wank et al. 2004a) and (2) as a functionalized surface to improve coupling of BN particles to a resin while simultaneously maintaining high thermal conductivity (Ferguson et al. 2000a, b; Wank et al. 2004b). A number of other applications followed.

### EBCs for passivation

**Fig. 25:**
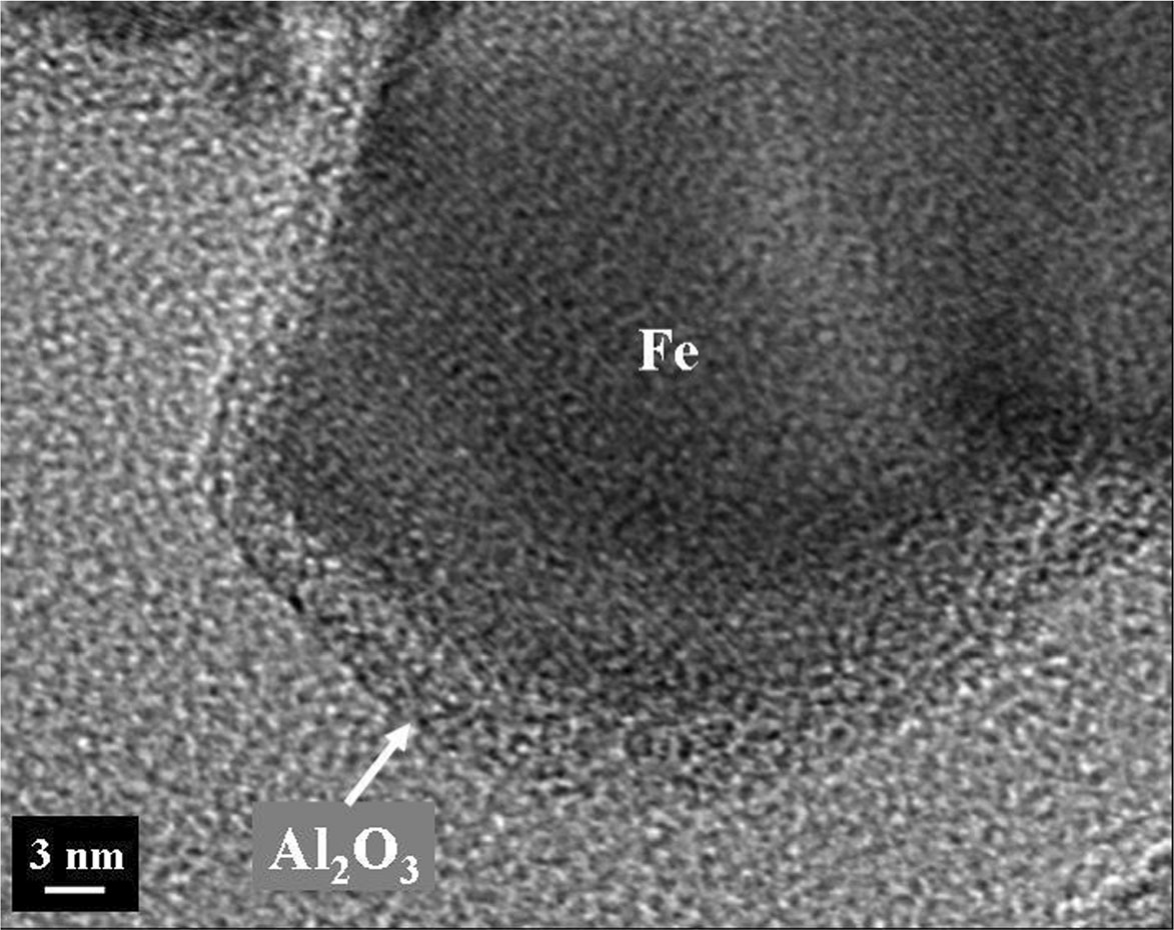
Cross-section high-resolution TEM (HRTEM) analysis of alumina-coated iron nanoparticles (synthesized in situ and immediately coated by particle ALD); composition verified by EDS (Hakim et al. 2007c)

**Fig. 26:**
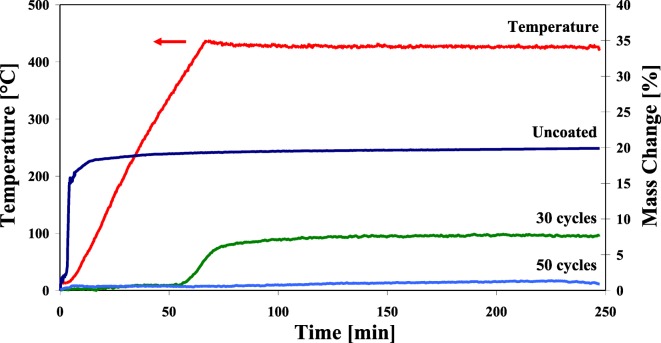
Oxidation resistance study of iron nanoparticles coated with Al_2_O_3_ after different numbers of TMA/H_2_O ALD coating cycles (Hakim et al. 2007c)

Magnetic nanoparticles are the focus of many exciting applications. These unique materials find use in areas, such as high-density data storage, magneto-optical switches, novel photoluminescent materials, biomedical diagnosis, catalysis, and environmental remediation, among others. A major limitation for extending the use of metal nanoparticles is their instability at ambient conditions. Owing to their very high surface area, metal nanoparticles spontaneously oxidize when exposed to air. While Wank et al. (2004a) showed that micron-sized iron particles could be passivated from oxidation at elevated temperatures with an ALD film, Hakim et al. (2007c) and King et al. (2009c) synthesized nanosized iron particles in situ from iron oxalate and then passivated them by alumina ALD in the same fluidized bed reactor. The synthesized 50 nm uncoated iron particles were pyrophoric when exposed to air at ambient temperature, whereas the particles coated with 30 ALD cycles (Fig. 25) were oxidation-resistant to 31 °C and particles having an 8 nm alumina film (50 ALD cycles) showed superior oxidation resistance at 427 °C (Fig. 26). The coated particles were ferromagnetic. Hence, nanosized metals can be synthesized and functionalized in situ prior to exposure to oxidizing atmospheres.

**Fig. 27:**
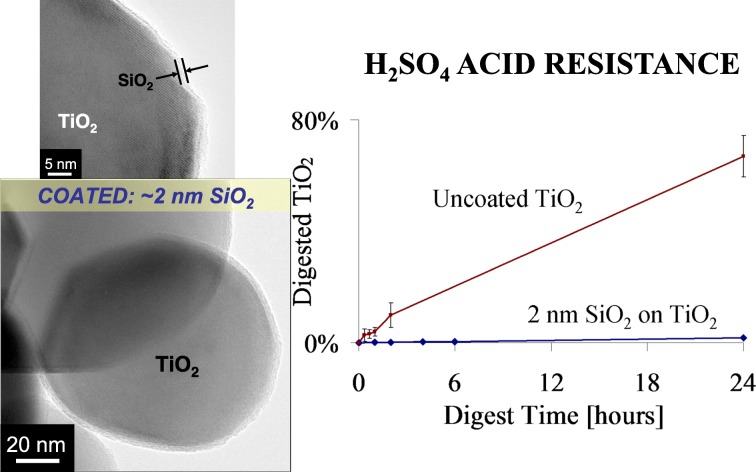
Silica on pigment grate TiO_2_. H_2_SO_4_ digest rate of pigment-grade anatase with and without 2 nm SiO_2_ films deposited by particle ALD (King et al. 2008b)

**Fig. 28:**
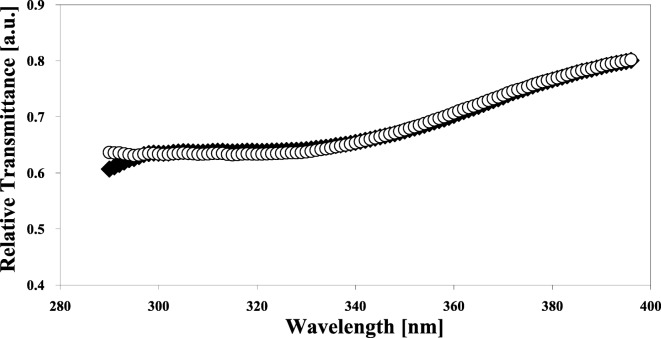
Relative UV-light transmittance of (♦) uncoated and (O) particle ALD alumina-coated titania nanoparticles (Hakim et al. 2007b)

Titanium dioxide (TiO_2_) is one of the most widely used white pigments in industrial applications, primarily due to its high refractive index, tinting strength, chemical inertness, thermal stability, non-toxicity, and inexpensive manufacture (Allen et al. 1992). The use of TiO_2_ pigments in the industry often involves the incorporation of the pigment into polymeric materials, such as paints and polyolefins. However, TiO_2_ is a well-known UV-activated oxidation catalyst, which degrades the polymer surrounding the pigment, through the so-called “chalking” process (Kemp and McIntyre 2006). Surface passivation is required for pigment-grade TiO_2_ particles used in commercial applications in order to quench the photocatalytic activity of the substrate material. Thin ALD films can be used to coat primary TiO_2_ particles with EBCs (Hakim et al. 2006, 2007b; King et al. 2008b, c, d, e; Liang and Weimer 2010). Pigment-grade TiO_2_ particles were passivated using nanothick insulating films fabricated by ALD (King et al. 2008b, c, d, e). Conformal SiO_2_ and Al_2_O_3_ layers were coated onto anatase and rutile powders in a fluidized bed reactor. SiO_2_ films were deposited using tris-dimethylaminosilane (TDMAS) and H_2_O_2_ at 500 °C. Trimethylaluminum and water were used as precursors for Al_2_O_3_ ALD at 177 °C. The photocatalytic activity of anatase pigment-grade TiO_2_ was decreased by 98% after the deposition of 2 nm SiO_2_ films. H_2_SO_4_ digest tests were performed to exhibit the pinhole-free nature of the coatings, and the TiO_2_ digest rate was 40 times faster for uncoated TiO_2_ than SiO_2_ coated over a 24 h period (Fig. 27). This is a significant result since film porosity is an attribute that directly correlates to a decrease in core material lifetime, specifically with respect to acid resistance for use in toners. Mass spectrometry was used to monitor reaction progress and allowed for dosing time optimization. These results demonstrated that the TDMAS-H_2_O_2_ chemistry can deposit high-quality, fully dense SiO_2_ films on high radius of curvature substrates. Particle ALD is a viable passivation method for pigment-grade TiO_2_ particles. Further, Hakim et al. (2007b) showed that Al_2_O_3_ ALD films (50 ALD coating cycles) on nano-TiO_2_ particles eliminated the photocatalytic activity of TiO_2_ nanoparticles, while maintaining their original extinction efficiency of ultraviolet light (Fig. 28) over the entire UV spectrum.

Recently, Hoskins et al. (2018) have shown that an Al_2_O_3_ or mullite (3Al_2_O_3_:2SiO_2_) coating on SiC can reduce steam oxidation of the SiC at 1000 °C (20 h) by up to 62% for a 10-nm-thick ALD film on micron-sized SiC particles. These results are comparable to CVD films that are three orders of magnitude thicker and support the conclusion that the superior ECB properties using ALD films is the result of the films being free of pin-holes. The mullite ECB is preferred since it has a thermal expansion coefficient similar to SiC. These results have substantial potential for ALD ECB coatings for passivation of SiC microchannel heat-exchanger surfaces.

Particle ALD ECBs may also mitigate problems with excess helium in spent nuclear fuel (Zhang et al. 2018). Helium gas accumulation from alpha decay during extended storage of spent fuel has potential to compromise the structural integrity the fuel. Zhang et al. (2018) reported results obtained with surrogate nickel particles which suggests that alumina formed by ALD can serve as a low-volume fraction, uniformly distributed phase for retention of helium generated in fuel particles, such as uranium oxide. Thin alumina layers may also form transport paths for helium in the fuel rod, which would otherwise be impermeable. Micron-scale nickel particles, representative of uranium oxide particles in their low helium solubility and compatibility with the alumina synthesis process, were homogeneously coated with alumina approximately 3–20 nm by particle ALD using a fluidized bed reactor. Particles were then loaded with helium at 800 °C in a tube furnace. Subsequent helium spectroscopy measurements showed that the alumina phase, or more likely a related nickel/alumina interface structure, retained helium at a density of at least 10^17^ atoms/cm^3^. High-resolution transmission electron microscopy (HRTEM) revealed that the thermal treatment increased the alumina thickness and generated additional porosity. Results from Monte Carlo simulations on amorphous alumina predicted that the helium retention concentration at room temperature could reach 10^21^ atoms/cm^3^ at 400 MPa, a pressure predicted by others to be developed in uranium oxide without an alumina secondary phase. This concentration is sufficient to eliminate bubble formation in the nuclear fuel for long-term storage scenarios, for example.

### Rheological behavior

Particle ALD can modify rheological behavior of nanoparticle suspensions (Hakim et al. 2007b) and of slurries and bulk powders comprised of 1 to 5 μm diameter particles (Kilbury et al. 2012). Microfine zinc powders, similar to those used in alkaline batteries, have been coated using boron nitride (BN) ALD films of sub-nanometer thickness or about 0.1 wt.%. The low-surface energy coatings reduced the cohesion of 1–5 μm particles by 52%. A highly loaded slurry of the same material in concentrated KOH showed a 10–30% reduction in slurry viscosity over a range of shear rates, with a shear thinning effect at high shear rates. Boron nitride (BN) platelets were coated using Al_2_O_3_ and SiO_2_ films to change the surface properties from hydrophobic to hydrophilic. As noted previously by Ferguson et al., the platelet structure of the BN provided for reactive surface functional groups on the edges while the basal planes only had an electron pair associated with nitrogen. Hence, while it was possible to coat the entire BN particle with Al_2_O_3_, a SiO_2_ coating was “patchy” and primarily on reactive edges. The coated and uncoated powders were dispersed into an epoxy to evaluate the solids loading to viscosity ratio. The ALD films improved the particle–resin adhesion and decreased the viscosity of an equivalently loaded slurry of uncoated powder (Fig. 8). Viscosity was reduced the most when the entire particle surface was coated by either Al_2_O_3_ or a SiO_2_/Al_2_O_3_ composite film. Coated microfine nickel, aluminum, and iron powders were also dispersed into epoxies, and lower viscosities and yield stresses were observed due to ceramic–epoxy interactions being more favorable than metallic–epoxy interactions.

**Fig. 29:**
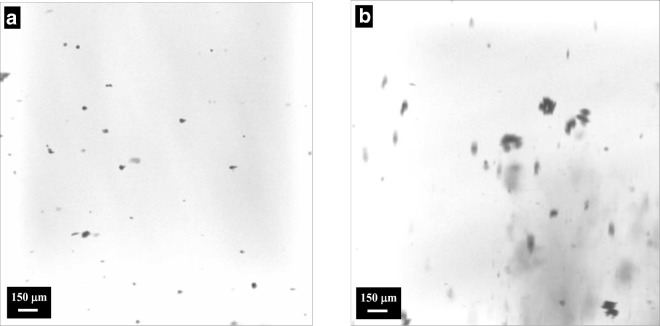
Fluidized aggregates of (**a**) uncoated and (**b**) particle ALD alumina-coated titania nanoparticles (Hakim et al. 2007a)

**Fig. 30:**
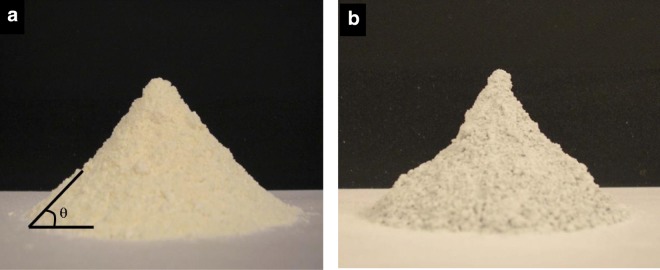
Images of (**a**) uncoated and (**b**) particle ALD alumina-coated titania nanoparticles utilized for angle of repose measurements (Hakim et al. 2007a)

**Fig. 31:**
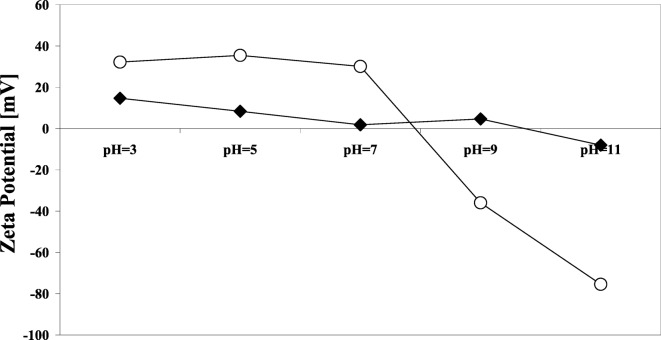
Zeta-potential analysis for titania nanoparticles before (♦) and after (○) particle ALD alumina coating (Hakim et al. 2007a)

The ALD platform can be used to modify surfaces of primary particles in order to change the interparticle and particle–liquid forces, which provides a lubricating effect without detracting from the bulk properties of the core particles themselves. Silica and titania nanoparticles were individually coated with ultrathin alumina films using atomic layer deposition (ALD) in a fluidized bed reactor. The effect of the coating on interparticle forces was studied (Hakim et al. 2007a). Coated particles showed increased interactions which impacted their flowability. This behavior was attributed to modifications of the Hamaker coefficient and the size of nanoparticles. Stronger interparticle forces translated into a larger mean aggregate size during fluidization (Fig. 29), which increased the minimum fluidization velocity. A lower bed expansion was observed for coated particles due to enhanced interparticle forces that increased the cohesive strength of the bed. Increased cohesiveness of coated powders was also determined through angle of repose (Fig. 30) and Hausner index measurements. The dispersability of nanopowders was studied through sedimentation and z-potential analysis. The optimum dispersion conditions and isoelectric point of nanoparticle suspensions changed due to the surface modification. The isoelectric point of titania particles changed from a pH of approximately 9.8 to a pH of about 7.9 after coating as shown in Fig. 31. This result further demonstrates that the ALD coating indeed modifies the interactions between particles in suspension. As an additional observation, the optimum dispersion conditions observed during sedimentation experiments (pH of 3 for uncoated and pH of 11 for coated particles) were also detected during zeta-potential analysis.

### UV-absorbing applications

Zinc oxide (ZnO) and TiO_2_ are wide (~ 3.3 eV) and medium (~ 3.0–3.2 eV) bandgap semi-conductor materials, respectively. They find use in a variety of optical, optoelectronic, and piezoelectronic applications, as well as in commodity markets, such as pigments, sunscreens, cosmetics, and even food products. TiO_2_ is also a well-known photocatalyst with a large propensity to photodegrade surrounding media because of free-radical generation in the presence of UV-light irradiation. Particle ALD applications using the UV-absorbing properties of ZnO and TiO_2_ include UV-driven water purification (King et al. 2009c; Zhou et al. 2010) and sunscreen/personal care (King et al. 2008b, c, d, e) products.

**Fig. 32:**
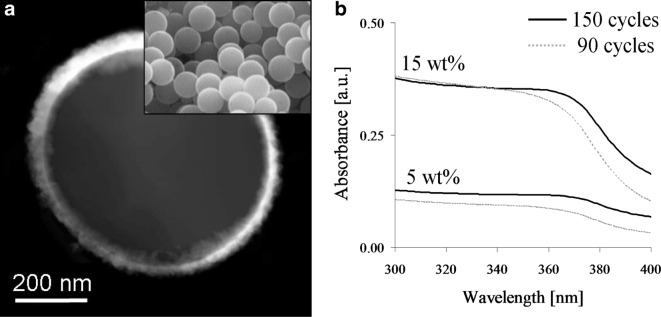
**a** STEM of 550 nm SiO_2_ sphere coated with 150 DEZ-H2O cycles and an SEM (inset) image of the uncoated substrate. **b** UV absorbance of dispersed SiO_2_ spheres coated with 90 and 150 ZnO cycles. The equivalent loading of uncoated SiO_2_ is used as the baseline for each. Scattering is neglected using this method (King et al. 2008c)

ZnO and TiO_2_ have been deposited on 550 nm SiO_2_ particle substrates using fluidized bed reactors. Diethylzinc (DEZ) and water were used as precursors at 177 °C (King et al. 2008b, c, d, e). Observed growth rates were ca. 2.0Å/cycle on primary particles as verified by HRTEM. Layers of 6 (30 ALD cycles), 18 (90 ALD cycles), and 30 (150 ALD cycles) nm were deposited for UV blocking cosmetic particles. The ZnO layers were polycrystalline as deposited. A scanning transmission electron microscopy (STEM) image is shown in Fig. 32a, in which ZnO appears white. The surface area of the SiO_2_ spheres was (5.6 ± 0.3) m^2^/g before and (5.7 ± 0.3) m^2^/g after coating with 30 ZnO cycles. This demonstrates the non-agglomerated nature of the final coated powder. The coated SiO_2_ particles were each dispersed at various solid loadings in mineral oil, and the diffuse UV transmittance was measured using an integrating sphere analyzer (Fig. 32b). The diffuse transmittance technique neglects scattering in this configuration, which allows for a direct observation of the differences in UV absorbance for changing film thickness and surface concentrations. The absorbance scales linearly with UV blocking content present at a given film thickness. At higher loadings (up to 25 wt%), the efficiency continues to increase but at a reduced rate.

**Fig. 33:**
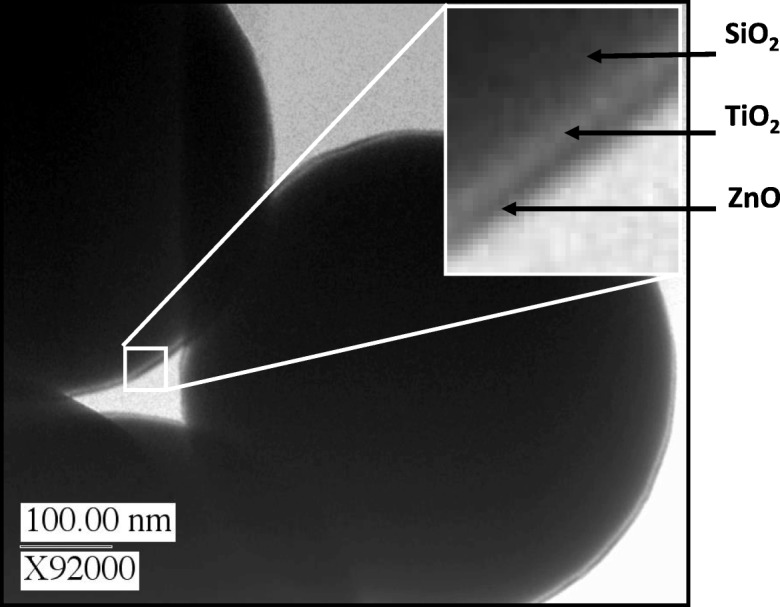
STEM of 550 nm SiO_2_ sphere coated by particle ALD with a multi-film of ZnO (UV-A blocker), TiO_2_ (UV-B blocker), and SiO_2_ (capping layer). Useful for providing “soft focus” sun protection factor (SPF) films and passivating layer to prevent contact with human skin

Dual ZnO/TiO_2_ films were placed on spherical SiO_2_ particles used in the cosmetics industry to effuse visible light and decrease the visibility of underlying features. TiO_2_ and ZnO coatings on these particles can enhance the visible light scattering power while simultaneously providing UV protection to the user. This dual coating is beneficial for fabricating superior sunscreen composite particles by combining the optical properties of the core and multi-layer shell materials. UVB rays (λ = 290–320 nm) are absorbed by the outer epidermal layers of human skin and are the cause of sunburn; UVA light (λ = 320–400 nm) has longer wavelengths and penetrates deeper into the skin and has recently been attributed to the predominant cause of skin cancer. The sun protection factor (SPF) is a weighted average calculation that predicts UV protection, but it is skewed far into the UVB spectrum. SPF is a good indicator of how well a sunscreen material will prevent sunburn but does not directly predict skin cancer prevention. ZnO nanoparticles are efficient UVA absorbers, but they do not produce high SPF sunscreen materials at typical loadings (2–5 wt.%). TiO_2_ films on ZnO nanoparticles can enhance the UVA absorbing core by increasing the propensity to scatter UVB light from a higher refractive index (RI) composite particle surface. These broadband UV blockers are photostable inorganic materials that can immediately be integrated into commercial sunscreen products. The development of a method to deposit dual ZnO/TiO_2_ films on particle substrates is desirable to be able to functionalize core particles with this versatile semiconductor material. TEM/HRTEM analyses were used to verify the presence of conformal bi-layer films on individual particle surfaces (Fig. 33). The TiO_2_ was successfully deposited using titanium tetraisopropoxide (TTIP) and H_2_O at 275 °C (King et al. 2008b, c, d, e). It was important to maintain operating temperatures below the thermal decomposition temperature of TTIP, which has been reported to begin near 300 °C. TTIP–H_2_O cycles at 100, 175, 250, and 325 were used in this study. The relatively low SA contributed to dose/purge times of 30/90 s and 60/120 s for TTIP and H_2_O, respectively.

The benefits of surface photocatalysts can be integrated with known magnetic separation techniques by creating photoactive magnetic particles (Zhou et al. 2010). Iron-based magnetic nanoparticles were produced by decomposition of iron oxalate powder, and then, a titanium dioxide (TiO_2_) thin film was deposited on the synthesized iron nanoparticles with an in situ atomic layer deposition (ALD) process at 100 °C using TiCl_4_ and H_2_O_2_ as precursors. However, because of the high surface area, the iron nanoparticles were unstable and spontaneously oxidized when exposed to H_2_O_2_ during the TiO_2_ ALD process, thus reducing the magnetic moment of the core particles. As an improvement in the process, prior to the TiO_2_ deposition, an aluminum nitride (AlN) film was deposited in situ to coat and passivate the iron core particles. The AlN ALD was performed at 250 °C with trimethylaluminum (TMA) and ammonia (NH3) as precursors. This passivation provided a significant decrease in the iron oxidation as determined by X-ray diffraction and magnetization measurements. Photoactivity of the TiO_2_ film was demonstrated by decomposition of methylene blue solution under ultraviolet irradiation.

### Quantum confinement

**Fig. 34:**
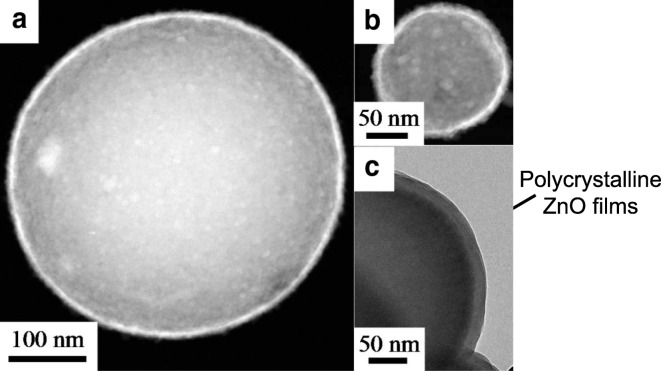
Deposited ALD ZnO nanoshells exhibiting 3D quantum confinement: (**a**) STEM image of a ZnO-coated 550 nm SiO_2_ sphere; (**b**) STEM image of a ZnO-coated 100 nm SiO_2_ sphere; (**c**) TEM image of a ZnO-coated 250 nm SiO_2_ sphere (King et al. 2009b)

**Fig. 35:**
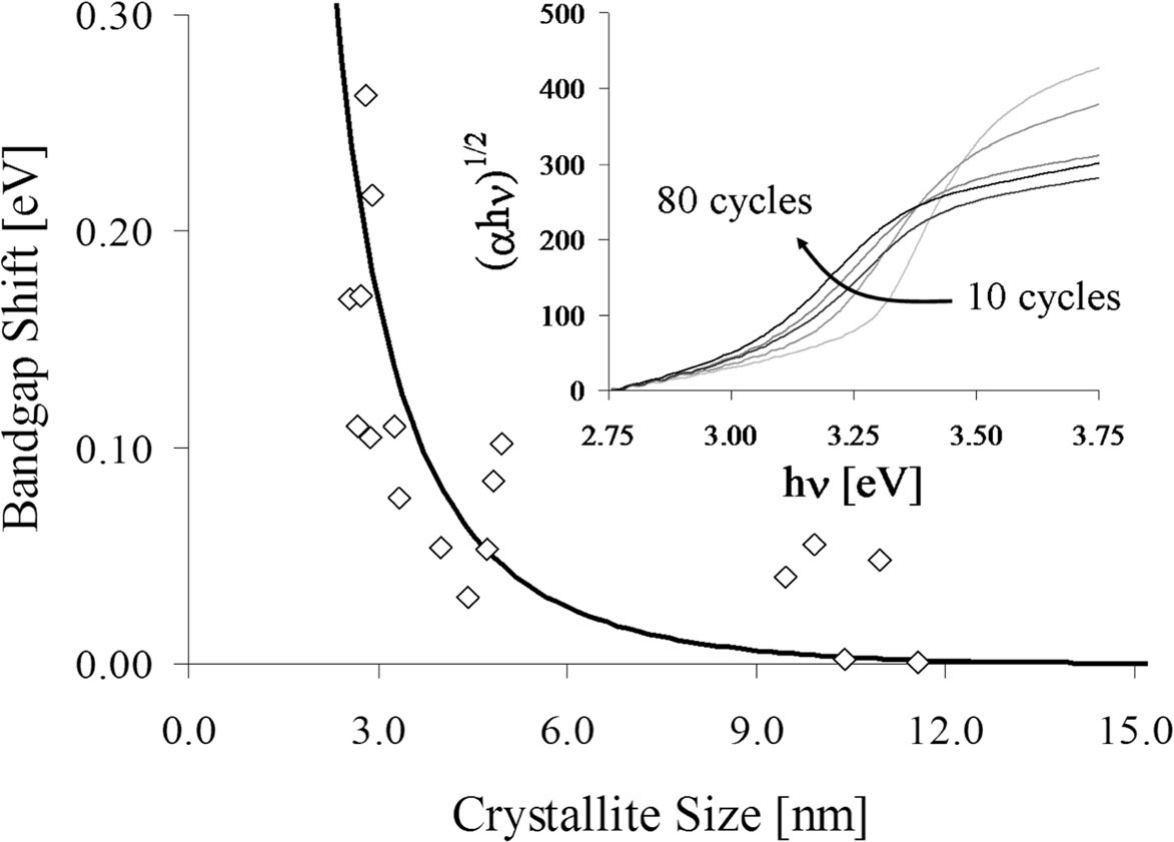
Quantum-confined bandgap shift across all ZnO ALD films in the experimental matrices with respect to the experimentally measured crystallite size of each. Inset image represents measurement geometry; the solid line is the predicted shift based on the Brus model for ZnO (King et al. 2009b)

Quantum confinement (QC) is an attribute that only nanoscale materials can possess and typically leads to remarkable optical, thermal, and electron transport properties that are significantly different than those of the bulk counterparts (Nozik 2008). The most common materials exhibiting QC are quantum dots, which are typically fabricated in liquid solutions or in the gas phase via molecular beam epitaxy (Bhattacharya et al. 2004). The non-linear optical absorption and emission properties of semiconductor quantum dots can be beneficial for a variety of applications and result in an increased opportunity for the nanoengineering of optoelectronic devices. Multiple exciton generation per absorbed photon has been demonstrated in quantum dot solar cells, leading to quantum yield efficiencies in excess of 250% (Beard et al. 2007). ALD can yield conformal, pinhole-free TiO_2_ films with blue-shifted absorbance properties in film thicknesses less than 10 nm (King et al. 2008a, 2009a). A blue-shift in absorbance corresponds to an increase in bandgap relative to the bulk, *E*g, bulk, which is a measure of the separation between the valence and conduction band energy levels of a material. The evolution of the crystal phase of quantum confined polycrystalline ZnO films fabricated by atomic layer deposition (ALD) was studied on spherical particle surfaces (King et al. 2009b) (Fig. 34). The factors of interest were the number of ALD cycles, the core particle size, and the use of post-deposition annealing temperatures up to 550 °C. The crystallite size of each peak increased almost linearly with the number of cycles and was further increased via thermal annealing steps. The shift in the optical bandgap of ZnO nanoshells was correlated to the domain size within the films at each point in the experimental matrix. The blue shift of 0.3 eV dissipated beyond crystallite sizes exceeding ∼ 10 nm, which was indicative of the successful deposition of quantum-confined nanostructures (Fig. 35). Grain growth shifts the bandgap according to the Brus model (Brus 1983), i.e., experimental validation of quantum confinement. An FBR or a high-throughput particle-coating reactor apparatus can be used to coat bulk quantities of particles with coatings that contain quantum-confined domains. The precision control capability of the ALD technique, coupled with the ability to predict crystallite size for given growth conditions, allows for tunable bandgap nanomaterials that could prove to be suitable for next-generation photovoltaic, thermoelectric, and/or optoelectronic devices.

### Catalyst stabilizers

Many catalytic processes, such as catalytic combustion, steam reforming, and automobile exhaust control, have reaction temperatures typically in excess of 300 °C. Metals are dispersed on high-surface area supports so that the resulting metal nanoparticles have a high fraction of their atoms on the surface. Catalysts can be designed using particle ALD (O’Neill et al. 2015). However, supported metal catalysts deactivate at high temperatures when these metal particles sinter to form larger particles. Particle ALD is used to reduce sintering (Feng et al. 2011; Liang et al. 2011; Lu et al. 2012; Kim et al. 2018; Phaahlamohlaka et al. 2018).

**Fig. 36:**
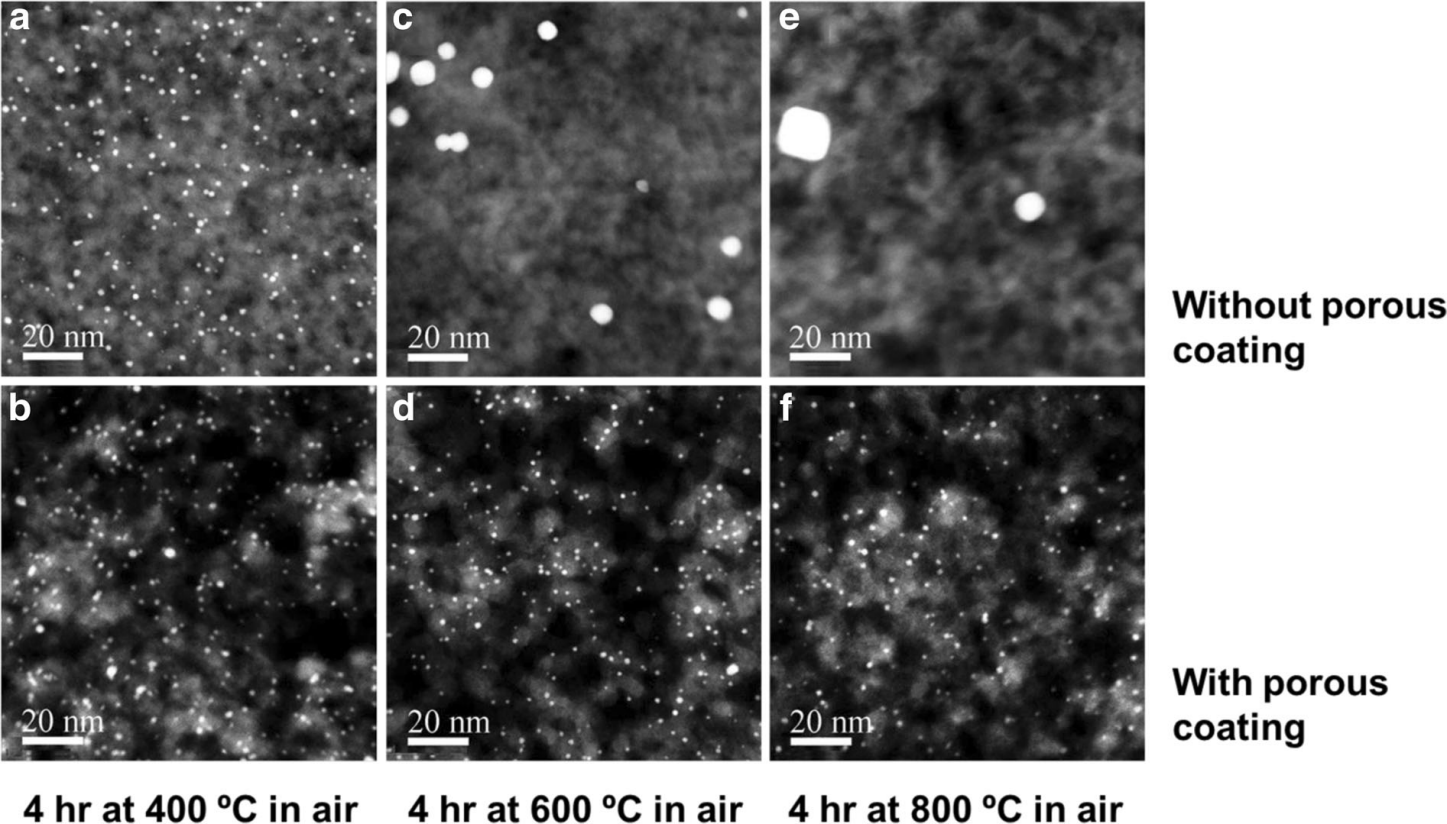
Cross-sectional STEM images of Pt/silica catalysts and porous alumina coated Pt/silica (40 MLD cycles) after calcination for 4 h at (**a**, **b**) 400 °C, (**c**, **d**) 600 °C, and (**e**, **f**) 800 °C (Liang et al. 2011)

Liang et al. (2011) demonstrated that supported Pt nanoparticles (< 2 nm) were stabilized by a highly porous, alumina nanofilm that was deposited on the Pt and its high-surface area silica support. The alumina film with sub-nanometer-thickness control resulted from thermal decomposition of an aluminum alkoxide layer that was deposited by molecular layer deposition (MLD). A catalyst with a porous ultrathin alumina layer was much more stable to calcination in air, even at 800 °C. Scanning transmission electron microscopy (STEM) images of the original and alumina-coated catalysts after heat treatment (Fig. 36) show that the original catalyst sinters much more than the alumina-coated catalyst at the higher temperatures. The particle sizes of the original catalyst do not appear to change at 400 °C for 4 h (Pt dispersion decreases slightly from 65 to 59%), but they increase significantly after treatment at 600 °C (Fig. 36c) (Pt dispersion decreases to 12% for 4 h). After treatment at 800 °C for 4 h, only two large Pt particles are seen over a large area (Fig. 36e) and Pt dispersion decreased to 3.9%. In contrast, the Pt particle size does not appear to change significantly for catalysts with 40MLD cycles, in agreement with the H_2_ chemisorption Pt dispersion measurements.

**Fig. 37:**
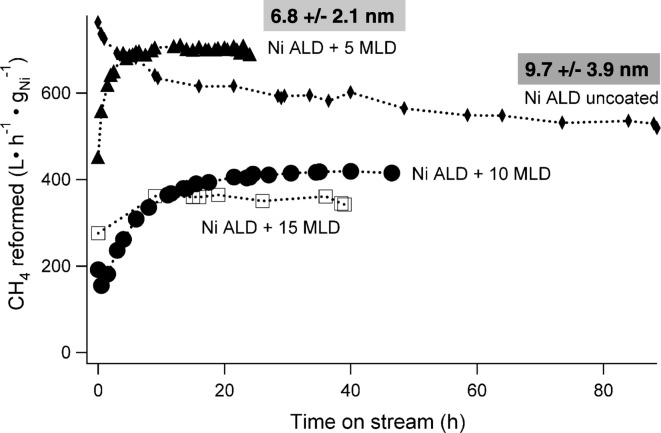
Molecular layer deposition (MLD) layers cause activation and stabilization during dry reforming of methane (DRM) rates at 700 °C for uncoated Ni ALD catalyst and the same catalyst coated with 5, 10, and 15 MLD layers (Gould et al. 2014)

To inhibit sintering of ∼ 5 nm-supported Ni particles during dry reforming of methane (DRM), Gould et al. stabilized catalyst with porous alumina grown by ABC alucone molecular layer deposition (MLD) (Gould et al. 2014). The uncoated catalyst continuously deactivated during DRM at 700 °C. In contrast, the DRM rates for the MLD-coated catalysts initially increased before stabilizing, consistent with an increase in the exposed nickel surface area with exposure to high temperatures. Post-reaction particles were smaller for the MLD-coated catalysts. Catalysts with only 5 MLD layers had higher DRM rates than the uncoated catalyst, and a sample with 10 MLD layers remained stable for 108 h. The DRM rates at 700 °C for uncoated Ni ALD catalyst and the same catalyst coated with 5, 10, and 15 MLD layers are shown in Fig. 37.

### Enhanced thermite materials

**Fig. 38:**
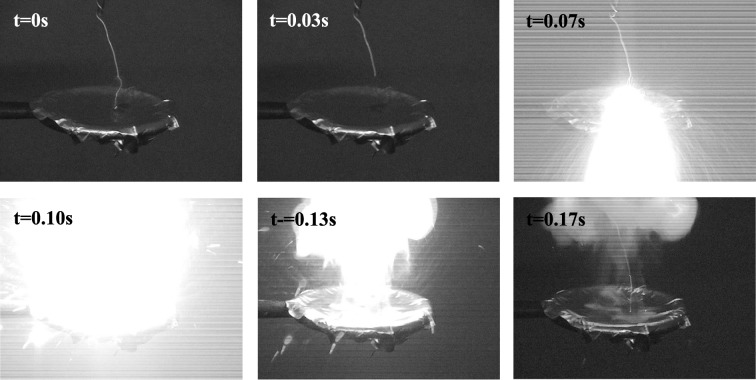
Sequential frames obtained from the ignition of SnO_2_-coated Al nanoparticles. The frames were obtained using a digital video recorder with a rate of 30 frames/s (Ferguson et al. 2005)

Thermite mixtures with improved contact between the fuel and oxidizer can provide increased reaction rates compared with the traditional thermite mixtures. One technique to create thermite mixtures with improved contact is to deposit the oxidizer directly onto nanometer-sized fuel particles. Ferguson et al. investigated the atomic layer deposition (ALD) of SnO_2_ onto nanoparticles using SnCl_4_ and H_2_O_2_ reactants at 250 °C (Ferguson et al. 2005). The nanoparticle ALD was performed in a small, hot wall, vertical fluidized bed reactor. SnO_2_ ALD was performed on Al nanoparticles. The SnO_2_-coated Al nanoparticles were ignited using a Tesla coil and filmed with a digital video recorder (Fig. 38). The detonation of the SnO_2_-coated Al particles produced a very bright flash. The detonation of the SnO_2_-coated Al particles was monitored and recorded with a digital camcorder. Six sequential frames from this detonation are displayed in Fig. 38. The frames were recorded at a rate of ~ 30 frames/s. Although the SnO_2_-coated Al particles were far from stoichiometric thermite composites, the SnO_2_-coated Al particles reacted much more quickly and violently than the uncoated Al particles. These results illustrate the utility of ALD techniques to coat oxidizers on fuel nanoparticles to create enhanced thermite materials.

### Ultrafast metal-insulator varistors

**Fig. 39:**
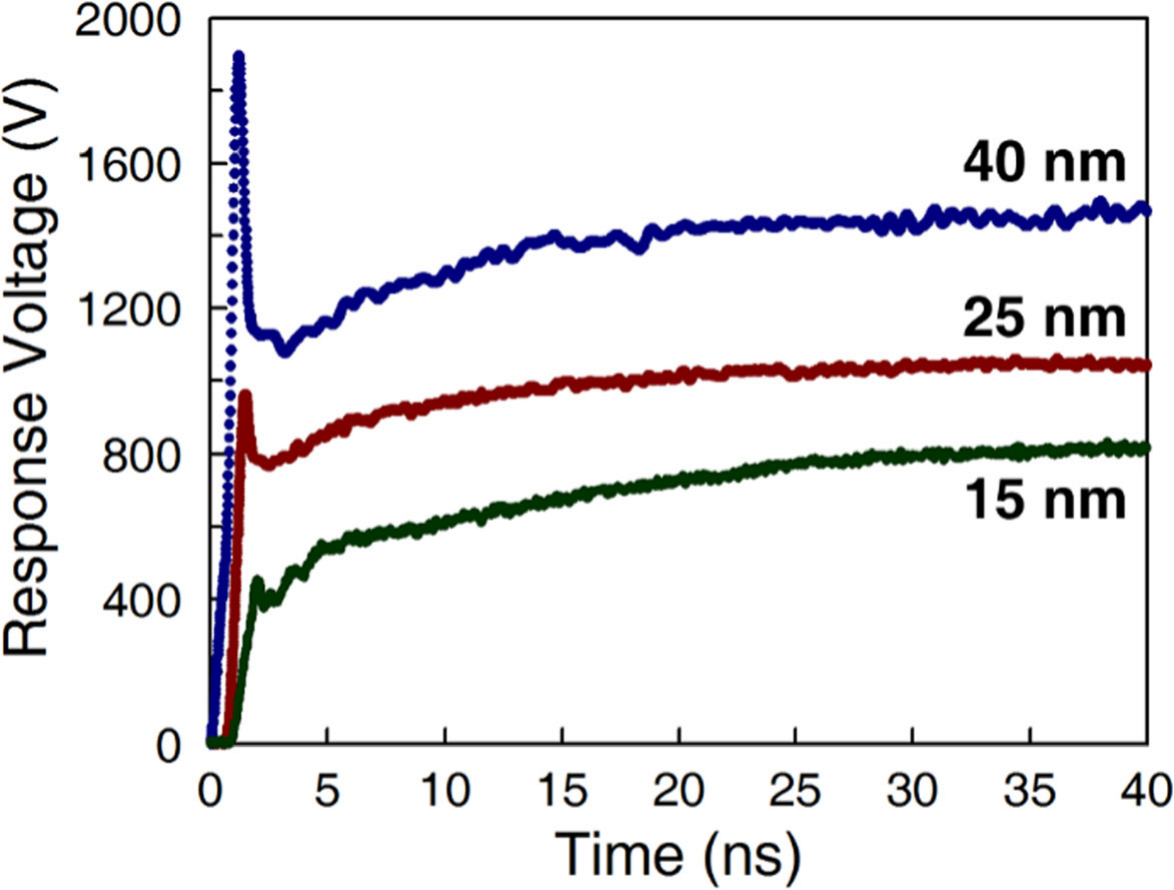
Typical Ni-based metal-insulator varistor responses to a 20 kV/0.7 ns rise-time transient for various Al_2_O_3_ tunnel junction thicknesses. The responses for the 15 and 25 nm thicknesses are shown offset in time by 1.0 and 0.75 ns, respectively, for clarity (Weimer et al. 2008a)

Varistors are non-Ohmic solid-state devices used for the suppression of electrical transients (Weimer et al. 2008b). Conventional metal oxide varistors (MOVs) are typically prepared by sintering ZnO grains in a flux of metal oxides, such as Bi_2_O_3_. Literature suggests that the conduction mechanism is a combination of thermionic emission in the pre-breakdown region and electron hole-assisted tunneling in the breakdown region. Due to the wide range of intergranular thicknesses produced by sintering and the heterogeneous structure of the sintered ceramic, MOVs are somewhat limited in their ability to exhibit extremely sharp non-Ohmic threshold behavior. Weimer et al. (2008a) fabricated metal-insulator varistors via atomic layer deposition (ALD) that exhibited significantly improved electrical properties including sub-nanosecond transient response times, low capacitances and leakages, and high non-linearities. A high-density matrix of micron-sized spherical Ni particles conformally coated with ~ 7.5–22 nm Al_2_O_3_ films exhibited transient response times (~ 0.3 ns), capacitances (~ 45 pF), leakage currents (~ 33 pA), and non-linearities (*α* ~ 380) which were all markedly improved over conventional metal oxide varistors. These characteristics result from the Fowler–Nordheim tunneling of electrons through uniform Al_2_O_3_ tunnel junctions separating adjacent particles within the matrix. The varistor clamping voltage was found to increase with Al_2_O_3_ tunnel junction thickness (Fig. 39). The clamping voltage could be precisely tuned by controlling the deposited Al_2_O_3_ thickness. These metal-insulator varistors exhibited transient response times, capacitances, leakage currents, and non-linearities that are markedly improved over conventional ZnO MOVs and may be useful for the suppression of fast-rise time transients.

### Solar thermochemical water and CO_2_ splitting active materials

**Fig. 40:**
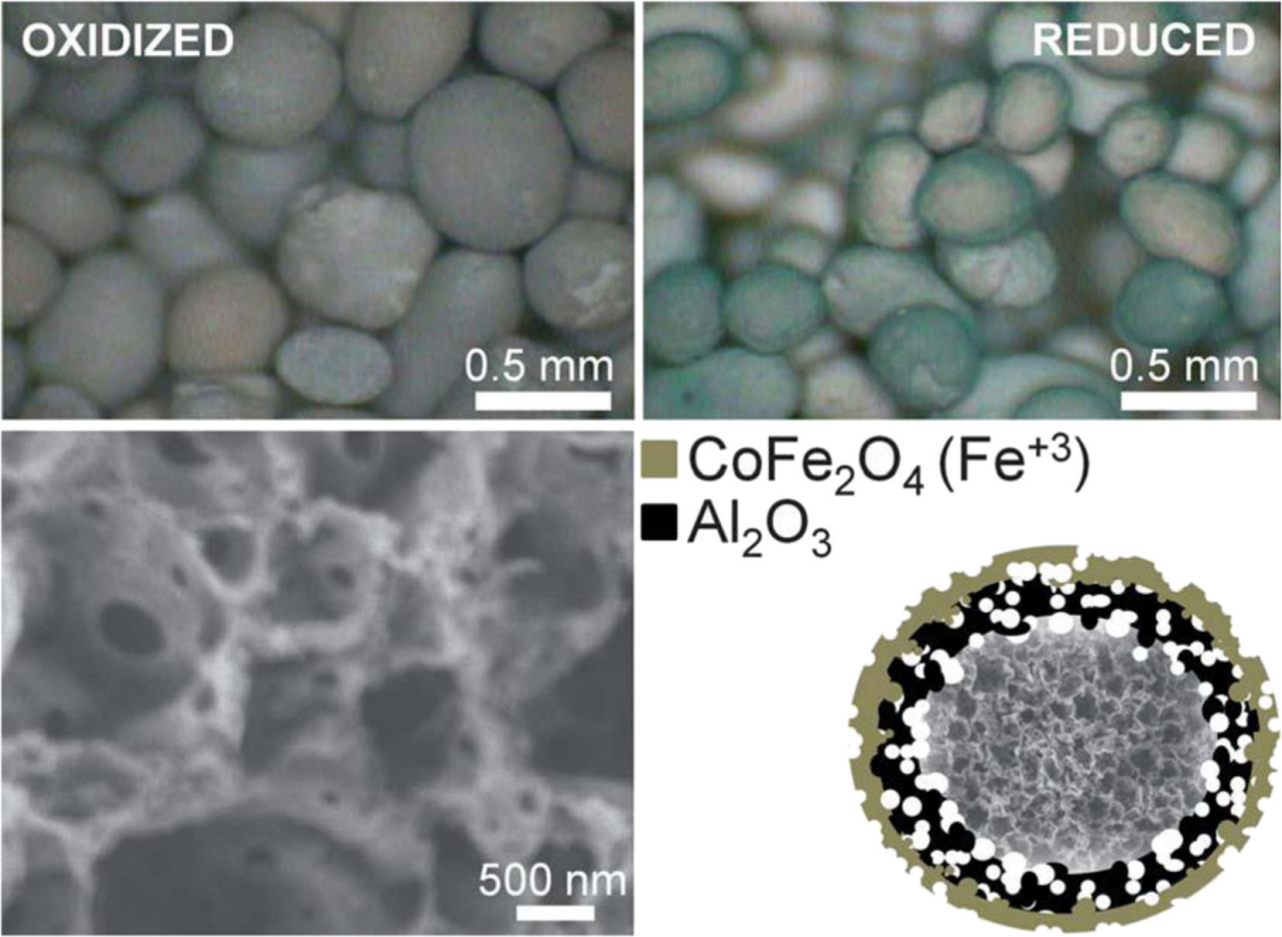
Optical image showing 0.5-mm-diameter spheroids of porous Al_2_O_3_ shells coated in nanometer-thick CoFe_2_O_4_. Color changes from brown to green when hercynite forms upon thermal reduction (top). FESEM image of the porous Al_2_O_3_ structure prepared by ALD (bottom left). Schematic illustrating the conceptual layout of the nanoengineered reactive structure, not drawn in the scale, and the spinel compound that forms upon calcination (bottom right). A representative FESEM image of the skeletal structure is incorporated into the schematic. The coverage of CoFe_2_O_4_ on the alumina scaffold is not limited to the outer surface; it coats all gas-accessible surfaces on and within the porous structure (Arifin et al. 2012)

**Fig. 41:**
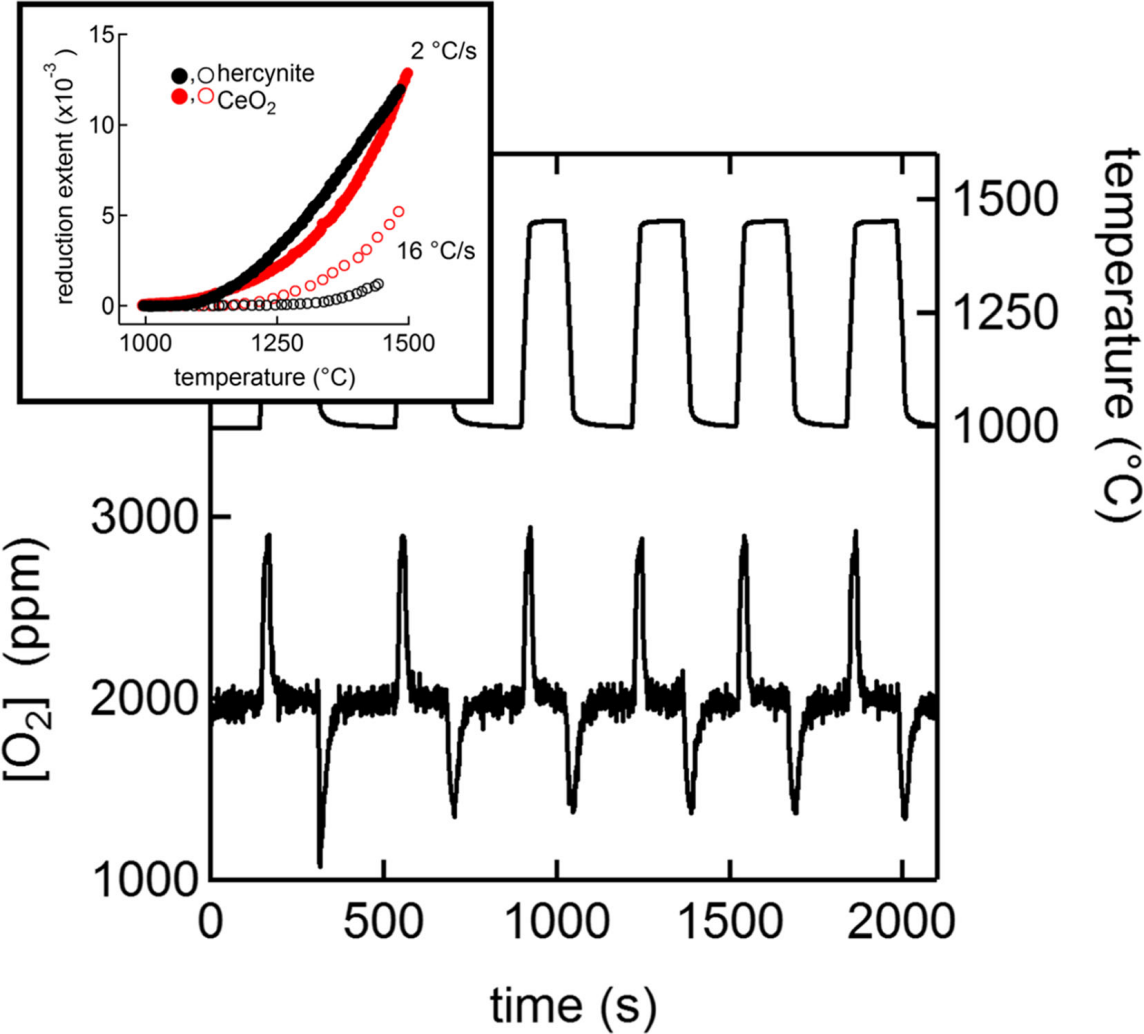
Oxygen uptake and release behavior as a function of time and temperature measured in the presence of a constant 2000 ppm O_2_ background partial pressure indicates that thermodynamics for hercynite reduction is favorable for solar-driven thermochemical cycles. The reduction extent as a function of temperature for ceria and hercynite at two different heating rates is shown in the inset (Arifin et al. 2012)

Particle ALD was used to fabricate cobalt-doped iron aluminate (hercynite) active materials for solar thermochemical redox CO_2_ and water splitting. Highly porous polymer particles were used as a template for alumina ALD prior to removing the polymer and leaving a skeletal alumina structure (Arifin et al. 2012) (Fig. 40). Iron oxide was prepared by ALD according to Scheffe et al. (2009). This processing allowed for only active material to be redox-cycled without an inert. The ALD-prepared engineered material maintained structural integrity over 6 heating cycles under conditions that mimic a concentrated solar power application, namely an oxidation temperature of 1000 °C, reduction at 1460 °C, and a heating rate of 16 °C/s from low to high temperature. Oxygen uptake and release behavior was similar to that of ceria, considered the state-of-the-art active material for solar thermochemical redox splitting (Fig. 41). This work demonstrated the efficacy of a completely different approach to two-step thermochemical CO_2_ splitting by blending novel chemistry with a nanoengineered reactive structure. The CoFe_2_O_4_-coated Al_2_O_3_ material was capable of producing appreciable amounts of CO after thermal reduction at a temperature as low as 1360 °C, with consistent oxidation behavior up to 23 thermal reductions. This observation was approximately 100 to 150 °C lower than values reported for ferrite- (Kodama and Gokon 2007) or CeO_2_- (Chueh et al. 2010) based systems, respectively. Adding compound-forming redox chemistries by ALD to the list of possible candidate material systems opened up new opportunities for discovery of better and more viable metal-oxide-based redox cycles for producing fuels from concentrated sunlight.

**Fig. 42:**
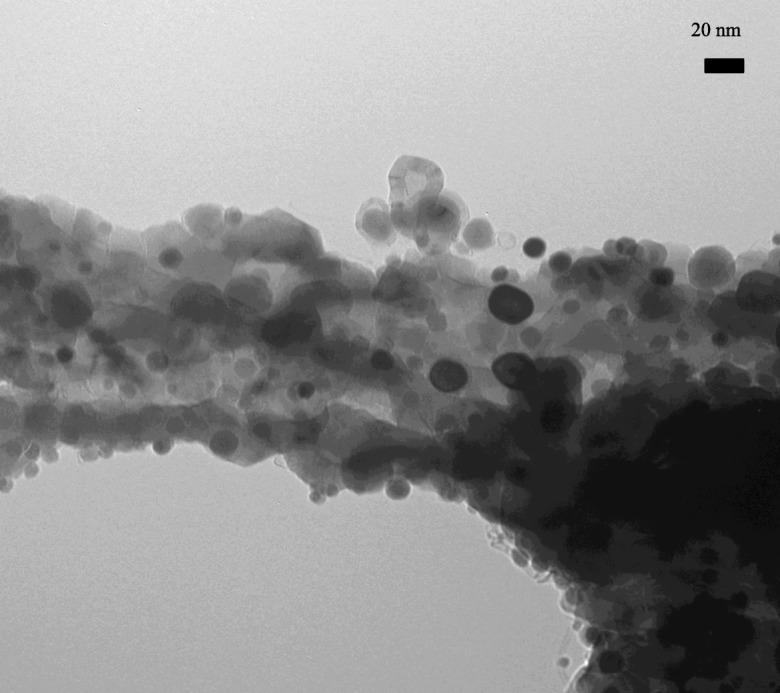
TEM of ALD-formed alumina structure with Co/Fe coating(cobalt-doped hercynite active material) (Lichty et al. 2012)

This ALD processing methodology was scaled-up by Lichty et al. (2012) and demonstrated on-sun using a thermochemical cavity receiver interfaced to a pilot-scale high flux solar furnace. Surface area measurements of cycled ALD particles showed improved surface area retention as compared to bulk Fe_2_O_3_ nanopowders. Reaction rates as high as 15.2 and 9.8 mmol/s/g were observed, on-sun, for H_2_O and CO_2_ splitting, respectively. Thermochemical cycling in a concentrated solar cavity reactor showed an order of magnitude increase in solar utilization efficiency between ALD particles and bulk Fe_2_O_3_ nanopowder. A transmission electron micrograph (TEM) of the ALD-prepared active Co-doped hercynite redox material is shown in Fig. 42.

### Li-ion battery materials

**Fig. 43:**
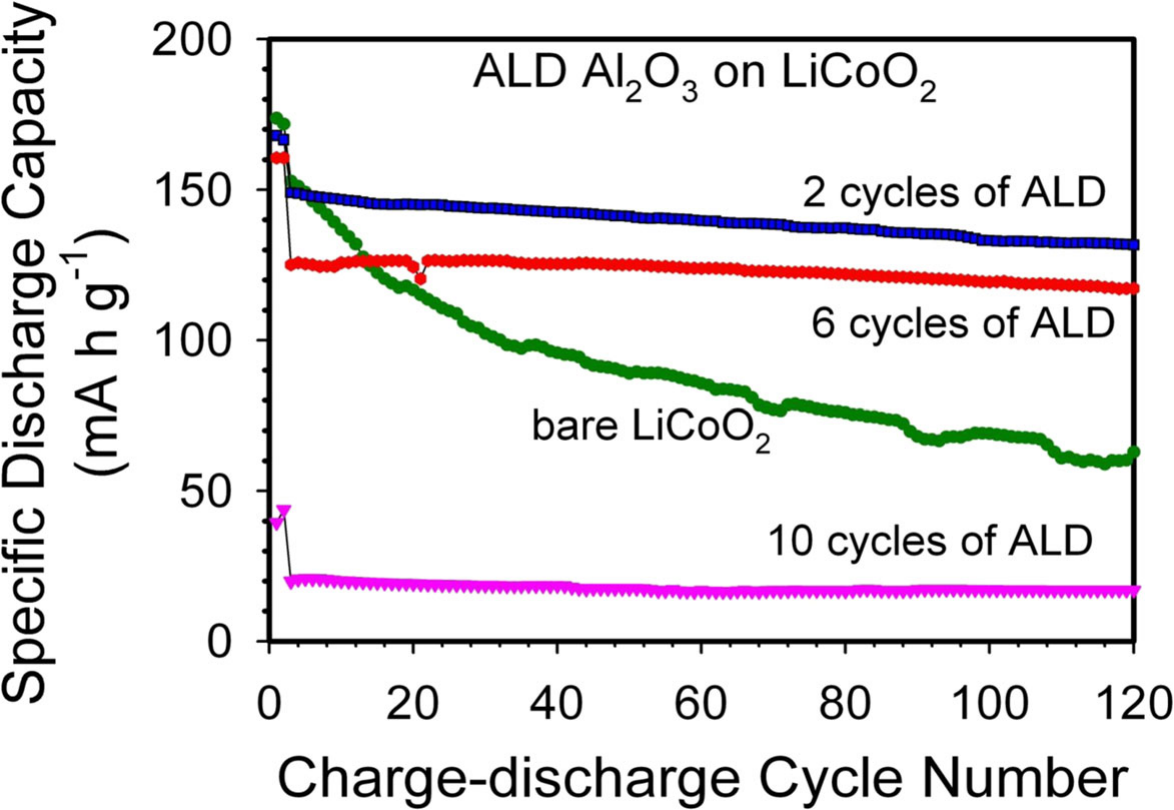
Particle ALD films improve LIB cathode performance. Charge–discharge cycle performance of electrodes fabricated using the bare LiCoO_2_ powders and the Al_2_O_3_ ALD-coated LiCoO_2_ powders using 2, 6, and 10 ALD cycles (Jung et al. 2010)

Lithium-ion batteries (LIBs) are very desirable because of their high-energy storage per volume and per mass. They have emerged as an important energy-storage device for portable electronics; however, they require higher stability for their use in plug-in hybrids or all-electric vehicles. LIB cathode materials typically have the composition of LiM_x_O_y_, where M is often Co, Ni, Mn, Fe, Al, Ti, or a combination of these metals. One of the most widely studied materials has been lithium cobalt oxide, i.e., Li_1 − *x*_CoO_2_, although there has been substantial recent interest in NCM (LiNi_x_Mn_y_Co_z_O_2_), i.e., lithium nickel cobalt manganese oxide. Cobalt dissolution, structural changes, and oxidative decomposition of the electrolyte produce a dramatic increase in the capacity fade at high-voltage potentials. These instabilities can be addressed by coating the LIB powders with metal oxide coatings, the majority of which have been based on solution techniques, such as the sol–gel method. These wet chemical coating methods require large amounts of solvent and precursor. A post-heat treatment is also necessary after the sol–gel coating. In contrast, ALD requires only a minimal amount of precursor, and ALD coatings are conformal and offer atomic-thickness control. The first substantive attempt to use an ALD coating for Li-ion batteries(Meng et al. 2012) was performed by Jung et al., where it was discovered that coating LiCoO_2_ powder with 2 cycles of Al_2_O_3_ ALD drastically improved capacity retention for half-cells charged to 4.5 V (Jung et al. 2010) as shown in Fig. 43. At this abnormally high charging potential, bare LiCoO_2_ degrades quickly. A coating of Al_2_O_3_ can prevent the LiCoO_2_ particles from decomposing electrolyte and forming a solid electrolyte interface (SEI) layer, but the films need to be extremely thin because Al_2_O_3_ is also an excellent insulator. Excellent capacity retention was observed for the LiCoO_2_ particles coated with only 2 ALD cycles (~ 0.25 nm thick, but likely a non-uniform coating due to nucleation (Puurunen 2005)), which represents *approximately one atomic layer of the Al*_*2*_*O*_*3*_*ALD coating*, while particles coated with 6 and 10 Al_2_O_3_ ALD cycles provide *lower* specific capacities. No other commercial process can apply such a thin coating to LIB particles.

**Fig. 44:**
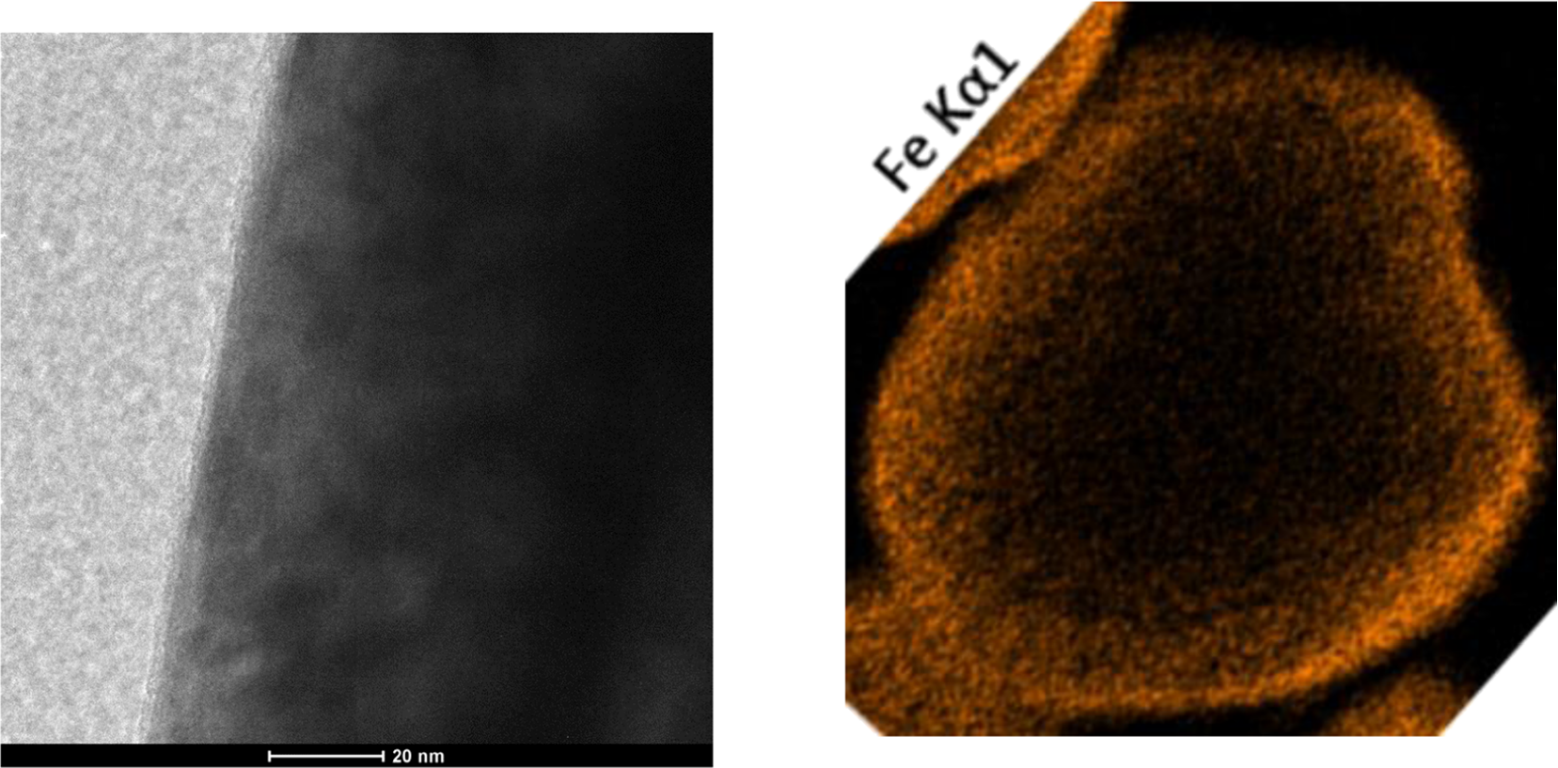
TEM images of (**a**) ~ 3 nm of conformal iron oxide film coated on one LiMn1.5Ni0.5O4 particle after 160 cycles of iron oxide ALD and (**b**) Fe element mapping of cross-sectioned surface by EDS; TEM image indicates that conformal iron oxide films were coated on primary LiMn1.5Ni0.5O4 particle surface. EDS mapping and EDS element line scanning indicates that Fe was doped in the lattice structure of LiMn1.5Ni0.5O4 (Patel et al. 2016b)

This work was extended to ion-conductive ALD films by Patel et al. (2016a, 2016b) in order to investigate a trade-off between the species transport (capacity) and protection (lifetime), resulting from the insulating properties of ALD films like Al_2_O_3_. They investigated ultrathin conformal cerium dioxide (CeO_2_) (Patel et al. 2016a) and iron oxide (FeO_x_) (Patel et al. 2016b) films on the surfaces of LiMn_2_O_4_ particles. The optimized CeO_2_ film-coated particles (~ 3 nm) exhibited a significant improvement in capacity and cycling performance compared to uncoated (UC), Al_2_O_3_ coated, and ZrO_2_ coated samples at room temperature and 55 °C for long cycling numbers. The initial capacity of the 3 nm CeO_2_-coated sample showed 24% increment compared to the capacity of the uncoated one, and 96 and 95% of the initial capacity are retained after 1000 cycles with 1 °C rate at room temperature and 55 °C, respectively. Detailed electrochemical data revealed that the suppression of the impedance rise and the facile transport of the species are the main contributors to the success for partial doping of iron (Patel et al. 2016b) on LiMn_1.5_Ni_0.5_O_4_ (LMNO) particles. The ionic Fe penetrates into the lattice structure of LMNO during the ALD process. Micrographs and a line scan for the Fe-doped LMNO materials are shown in Fig. 44. After the structural defects were saturated, iron started participating in the formation of ultrathin oxide films on LMNO particle surfaces. Owing to the conductive nature of iron oxide films, with an optimal film thickness of ~ 0.6 nm, the initial capacity improved by ~ 25% at room temperature and by ~ 26% at an elevated temperature of 55 °C at a 1 °C cycling rate. The synergy of doping of LMNO with iron combined with the conductive and protective nature of the optimal iron oxide film led to a high-capacity retention (~ 93% at room temperature and ~ 91% at 55 °C) even after 1000 cycles at a 1 °C cycling rate.

ALD’s applications have been extended to sodium-ion (Meng 2017a, b) batteries and lithium-sulfur (Sun et al. 2018) batteries. It is anticipated that particle ALD will play a key role in the low-cost coating of LIB cathode materials since preventing capacity fade is a key element to LIBs gaining widespread acceptance.

### Electroluminescent phosphors

Phosphors are used in flat-panel plasma displays (FPDs), cathode ray tubes, X-ray imaging devices, field emission devices, fluorescent lighting fixtures, and a variety of other applications to generate visual images or simply provide light. Although a wide variety of phosphor materials are known for use in these applications, those materials all have in common the ability to generate a characteristic light in response to exposure to an excitation energy source. The excitation energy source may be, for example, an applied electrical field (electroluminescence (EL)). Electroluminescent phosphors are of particular interest for flat-panel display applications. Flat panel displays commonly include a phosphor layer which is sandwiched between two insulator layers. The phosphor material is commonly printed as a thin film onto an adjacent layer. There are several reasons that make it preferable to use as a powdered phosphor. Chief among these is cost—powdered phosphors can be used in very small amounts and so the amount of phosphor that is needed can be significantly reduced. In addition, light loss through internal reflection can be minimized using particles and there is no loss in brightness due to light lost at edges, as in thin phosphor films. Efficiency (light emitted/unit applied power) is also higher for powders. The use of powders also makes it possible to produce all colors in a single phosphor plane, as a particulate mixture of different color-emitting phosphors can be formed as a single layer. The phosphor particles typically are composites of a host material that in the case of electroluminescent particles provides a necessary set of electrical properties and one or more “luminescent centers.” The “luminescent centers” are usually metal cations and sometimes anions which are “doped” or otherwise combined with the host material. These ions usually become incorporated into the crystalline lattice of the host material or dispersed as discrete domains within the host material. The luminescent centers provide the desired optical emission properties to the phosphor particles. Again, a wide variety of these materials is known, which differ in their composition according to the specific application and desired emitted color. Phosphors that emit white, yellow, red, green, and blue wavelengths of visible light are commonly used in display and monitor applications. It is often necessary to coat the surface of the phosphor particles. Reasons for doing this include (1) particle protection, often against reaction with water but also against reaction with air, other oxidants, or contaminants; (2) improving screening characteristics; and (3) improving contrast or pigmentation. Among the coating materials used for these purposes are ZnO, MgO, In_2_O_3_, Al_2_O_3_ and SiO_2_, and CuS. Chemical vapor deposition (CVD), sol-gel methods, and, most recently, particle ALD (Weimer et al. 2014) have been used to provide coatings of these types. To be effective, the applied coating needs to be as uniform and as thin as possible. It is also beneficial that the coating process does not cause individual particles to agglomerate to form larger aggregates. In addition to having much larger diameters than are wanted, these aggregates often tend to break apart, revealing defects in the coating at the break areas. The underlying particles are subject to attack from water, oxidants, and other materials at the places where these defects occur. Neither CVD nor sol-gel techniques are entirely satisfactory, as agglomerates tend to form readily in these processes. In addition, these methods require relatively large amounts of raw materials, as only a portion of the applied reactants actually become applied to the surface of the phosphor particles. Quite often, materials applied by these processes form separate particles instead of forming films on the surface of the phosphor particles. For various reasons, it is desired to develop phosphor particles that are smaller than those commonly used now. Commercially available phosphor particles usually have diameters in the 1–50 μm range. Phosphor particles having diameters of less than 1 μm, and in particular less than 100 nm, potentially offer advantages in screen design and performance. CVD and sol-gel coating methods are particularly unsuitable for coating these smaller particles. Particle ALD provides the opportunity for similar passivation as CVD, but with a substantially thinner film due to the conformal and pinhole-free nature of the ALD film (Table 1). Another advantage is similar brightness, but requiring less energy due to the much thinner film. The use of particle ALD for phosphor coating has tremendous commercial potential due to a substantial cost/performance benefit compared to CVD. It is clear that for lighting applications, such as LEDs, ALD will be the clear winner compared to other coating technologies.

**Table 1:** EL Phosphor CVD vs. ALD Barrier Layer Performance

Attribute	CVD-coated control	300 Al2O3 cycles	600 Al2O3 cycles
Initial brightness (%)	79.6	96.0	96.3
24 h (%)	73.8	89.5	90.0
100 h (%)	68.8	73.4	84.4
Maint. (%, 100 h)	86.3	76.4	87.6
Coating thickness (Å)	2080	320	800

Test conditions: 21 °C, 50% RH

ALD coatings provide equivalent or improved water/oxygen protection at lower film thicknesses and at higher device brightness; U.S. Patents 8,298,666 and 8,637,156

### Ceramic particle sintering aid additives

**Fig. 45:**
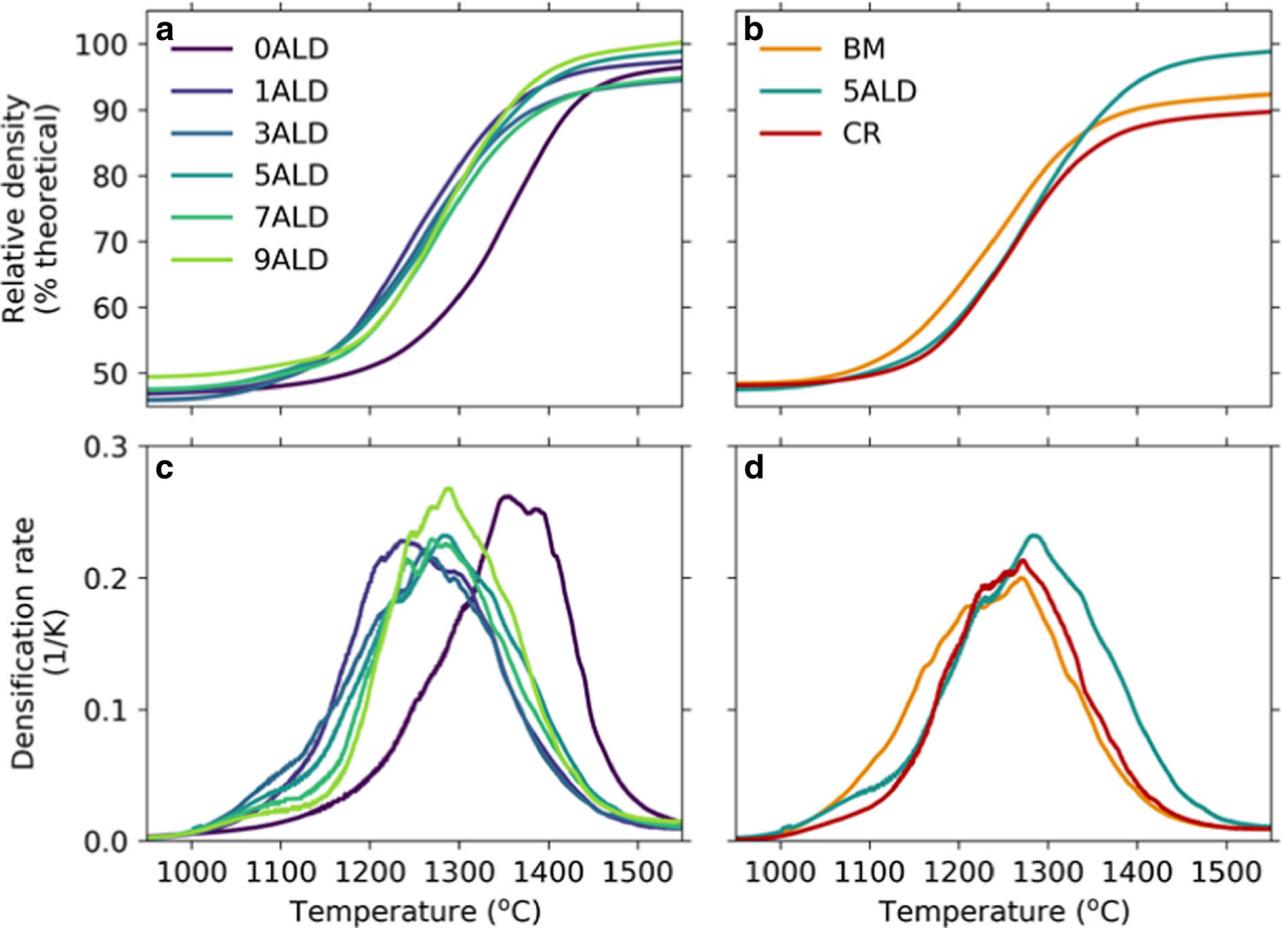
Relative density versus temperature for (**a**) 0–9ALD cycles and (**b**) 5ALD cycles (amorphous Al_2_O_3_ film), BM (ball-milled), and CR (crystalline Al_2_O_3_ film) for the 10 °C/min CRH (constant rate of heating) dilatometer experiment, and densification rate versus temperature for (**c**) 0–9ALD cycles and (**d**) 5ALD cycles, BM, and CR for the 10 °C/min CRH experiment (O’Toole et al. 2018)

**Fig. 46:**
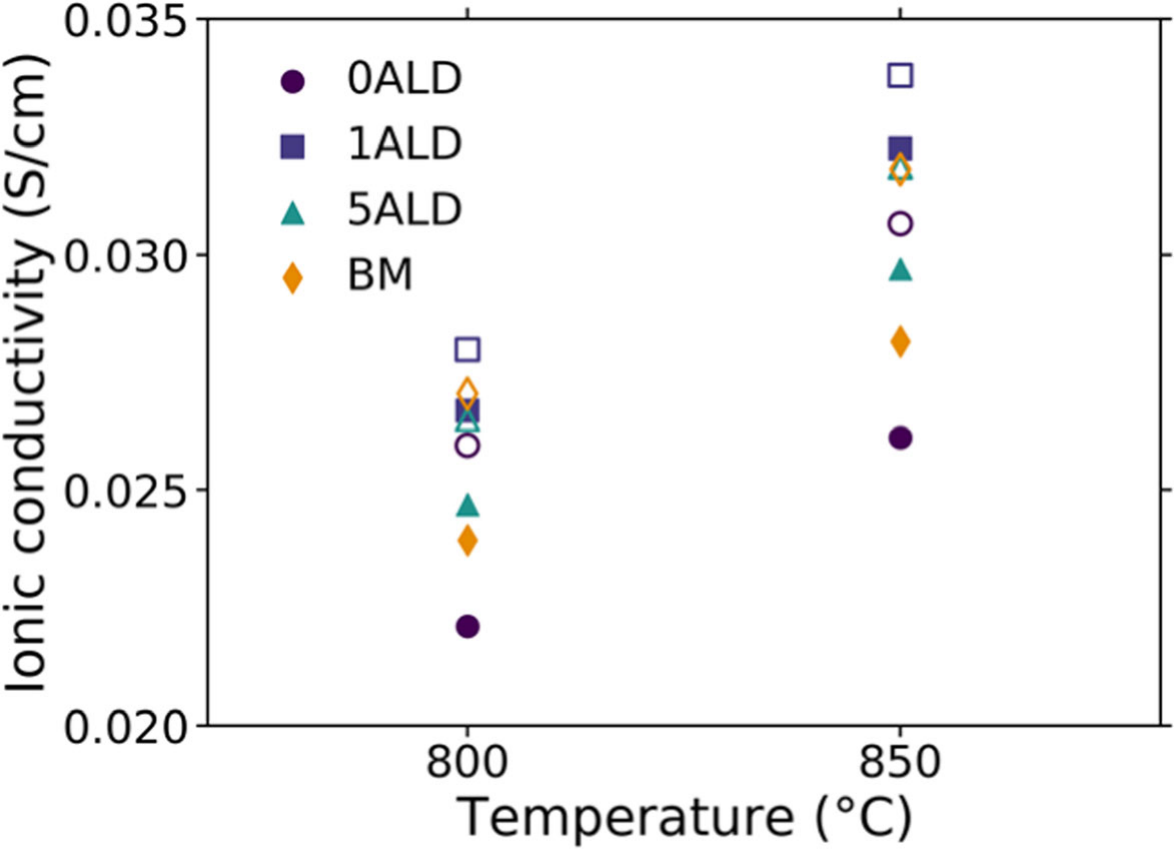
Average ionic conductivity measurements for 0ALD cycles, 1ALD cycle, 5ALD, cycles, and BM sintered at 1350 °C for 2 h, where close markers (●, ■, ▲, ♦) indicate measured ionic conductivity and open markers (○, □, ▯, ◊) indicate adjusted conductivity. The standard error for triplicate experiments was < 3% for all sample types and temperatures (O’Toole et al. 2018)

Sintering aids are incorporated into ceramic precursors to promote densification at reduced temperatures by changing the mechanism of sintering (e.g., solid-state to liquid-phase) and/or increasing the diffusion coefficient of migrating ions (German 1985; Boniecki et al. 2010). The dispersion of sintering aid within the primary ceramic is critical to the performance of the additive and is typically achieved using milling (Matsui et al. 2008a, b; Lei et al. 2014; Yu et al. 2016), spray drying, and/or colloidal processing (Wang and Raj 1990, 1991; Tekeli and Demir 2005; Suárez and Sakka 2010; Song et al. 2011). However, these methods can result in localized regions of excess or deficient sintering aid which can reduce the homogeneity of the final microstructure and adversely affect material properties (Hodgson et al. 1999; Matsui et al. 2008a, b). O’Toole et al. (2018) recently used particle ALD to precisely coat individual yttria-stabilized cubic zirconia (YSZ) precursor particles with a desired thickness of amorphous Al_2_O_3_. YSZ is an attractive oxygen ion conductor for use as an electrolyte in solid oxide fuel cells (SOFC) and other electrochemical devices (Singhal 2000; Stetter et al. 2003; Hui et al. 2007; Ebbesen and Mogensen 2009; Tao et al. 2016). The incorporation of small amounts of aluminum oxide (Al_2_O_3_) as a sintering aid decreases the sintering temperature required to reach near-theoretical density without deleteriously affecting key dense part properties, such as ionic conductivity (Mori et al. 1994; Feighery and Irvine 1999; Lee et al. 2000; Lei et al. 2014; Yu et al. 2016) and mechanical strength (Choi and Bansal 2005; Tekeli et al. 2008). Constant rate of heating (CRH) experiments were conducted for all YSZ sample types at four heating rates (5, 10, 15, and 20 °C/min) resulting in densification curves such as those shown in Fig. 45a, b for the 10 °C/min CRH experiments. The densification curves show that the presence of Al_2_O_3_ (at all concentrations evaluated) decreases the temperature at which densification begins (i.e., where the densification rate exceeds 0.05 K^−1^) by ~ 100 °C compared with the control sample (0ALD). Densification rate versus temperature curves are shown in Fig. 45c, d. Again, the presence of Al_2_O_3_ decreases the temperature at which the peak densification rate occurs by ~ 100 °C compared with the control sample. The addition of 0.7 wt% Al_2_O_3_ with one particle ALD cycle enhanced the ionic conductivity of YSZ by 23% after sintering at 1350 °C for 2 h (Fig. 46), demonstrating that dense parts with high oxygen ion conductivities can be produced after sintering at reduced temperatures. One particle ALD cycle is a fast, easily scaled-up process that eliminates the use of solvents, washing, filtering, drying, and deagglomerating and has substantial cost/performance advantages over conventional processing. It is anticipated that substantial future research will focus on using particle ALD for providing sintering aids to other substrate ceramic precursor powders.

## Perspectives, challenges, and path forward

Particle ALD allows the design and fabrication of complex atomic nanostructures using particulate precursors. Primary particles, including nanoparticles and high-aspect ratio nanotubes, can be coated if one uses an agitated processing system. Film thickness can vary for different applications and ranges from sub-nanometer to tens of nanometers thick. Films can be uniform or non-uniform depending upon the functionalization of the particle surfaces and the nucleation required for a given ALD chemistry. Particle ALD is a low-cost process due to the ability to use almost 100% of sequential precursors (Fig. 9). This recognition of low cost was a major stumbling block in the consideration and adaptation of particle ALD for commercial applications. Another major hurdle was convincing possible users that primary (individual) particles, including nanoparticles, could be coated without agglomeration (Hakim et al. 2005a, b). A major opportunity exists for developing continuous low-cost spatial particle ALD processes for huge tonnages of commercial products that require only a few ALD cycles, such as LIB materials. Low cost will require those continuous processes to efficiently use expensive precursors with near 100% usage similar to the efficiency of batch-fluidized beds. Further, because of the handling of such large quantities of fine powders reacting with sequential gases, those spatial ALD processes need to be relatively simple and to avoid being “solids processing nightmares” having major powder handling issues. While most applications have yet to be discovered, likely initial commercial products will employ particle ALD for cathode battery and lighting application material passivation, as well as catalyst sintering prevention and as sintering additives for advanced ceramic materials. Annual citations for particle ALD have grown exponentially with time from one citation in 1999 to 3933 citations in 2017, totaling more than 21,500 citations according to the Web of Science. Exponential interest in particle ALD is continuing.
